# Patient Preferences to Assess Value IN Gene Therapies: Protocol Development for the PAVING Study in Hemophilia

**DOI:** 10.3389/fmed.2021.595797

**Published:** 2021-03-09

**Authors:** Eline van Overbeeke, Brett Hauber, Sissel Michelsen, Michel Goldman, Steven Simoens, Isabelle Huys

**Affiliations:** ^1^Clinical Pharmacology and Pharmacotherapy, University of Leuven, Leuven, Belgium; ^2^Health Preference Assessment, RTI Health Solutions, Durham, NC, United States; ^3^Healthcare Management Centre, Vlerick Business School, Ghent, Belgium; ^4^Institute for Interdisciplinary Innovation in Healthcare, Université Libre de Bruxelles, Brussels, Belgium

**Keywords:** preference, instrument design, hemophilia, interviews, survey, gene therapy

## Abstract

**Introduction:** Gene therapies are innovative therapies that are increasingly being developed. However, health technology assessment (HTA) and payer decision making on these therapies is impeded by uncertainties, especially regarding long-term outcomes. Through measuring patient preferences regarding gene therapies, the importance of unique elements that go beyond health gain can be quantified and inform value assessments. We designed a study, namely the Patient preferences to Assess Value IN Gene therapies (PAVING) study, that can inform HTA and payers by investigating trade-offs that adult Belgian hemophilia A and B patients are willing to make when asked to choose between a standard of care and gene therapy.

**Methods and Analysis:** An eight-step approach was taken to establish the protocol for this study: (1) stated preference method selection, (2) initial attributes identification, (3) stakeholder (HTA and payer) needs identification, (4) patient relevant attributes and information needs identification, (5) level identification and choice task construction, (6) educational tool design, (7) survey integration, and (8) piloting and pretesting. In the end, a threshold technique survey was designed using the attributes “Annual bleeding rate,” “Chance to stop prophylaxis,” “Time that side effects have been studied,” and “Quality of Life.”

**Ethics and Dissemination:** The Medical Ethics Committee of UZ KU Leuven/Research approved the study. Results from the study will be presented to stakeholders and patients at conferences and in peer-reviewed journals. We hope that results from the PAVING study can inform decision makers on the acceptability of uncertainties and the value of gene therapies to patients.

## Introduction

The pharmaceutical sector is shifting from a focus on classic chemical and first-generation biological medicines to the development of more complex biological therapies like gene therapy. Gene therapies are high-cost treatments, but may come with the promise of permanent benefits or even a cure. First efforts to market European Medicine Agency (EMA) approved gene therapies showed that obtaining market access is difficult (1). One of the main challenges is that uncertainty on magnitude and duration of effect may limit value perceived by HTA and payers (1, 2). In this context, uncertainty regarding long-term efficacy and safety is caused by limited comparative data and lack of long-term evidence (1). With the rise of therapies that have the potential to create permanent effects in patients, decision-making on the macro (marketing authorization), meso (pricing and reimbursement), and micro (shared-decision making) level will increasingly have to deal with uncertainty regarding long-term efficacy and safety.

With regard to value assessments of therapies potentially offering a cure, it has been argued that Quality Adjusted Life Years (QALYs) may not be appropriate for use, may be insensitive and may not cover all aspects of gene therapies relevant to patients; possibly resulting in a misjudgment on the value of such therapies (3–5). Gutknecht et al. (4) stated that QALYs only reflect outcomes that have a direct impact on Quality of Life (QoL) and/or survival, and suggested that through measuring patient preferences also other treatment features (e.g., mode of administration and cost) can be considered.

Performing patient preference studies in the context of gene therapies will not take away the uncertainty regarding long-term outcomes that can only be resolved by life-long follow-up of these patients, and will most likely not replace use of QALYs as this measure allows for comparison across diseases. However, performing patient preference studies in this context can inform decision-making by providing (1) additional insights on the acceptability of uncertainties to patients, (2) insights on the value of these therapies to patients, and (3) a pathway for the patient to weigh in on decision-making regarding gene therapies.

One of the rare diseases for which gene therapies are in development is hemophilia (A and B) (6–8). Current hemophilia treatment consists of regular intravenous administration of factor replacement therapy. In hemophilia, unmet medical needs result from the invasiveness of current treatment, the fluctuations of achieved factor levels making patients more prone to bleeds and joint damage, and the development of antibodies against current therapies in some patients (9–12). In hemophilia, gene therapy comes with the promise that one infusion could potentially replace lifelong administration of other high-cost drugs. To date, no research has been conducted regarding the preferences of hemophilia patients regarding gene therapy (13).

Therefore, we decided to initiate the Patient preferences to Assess Value IN Gene therapies (PAVING) study, to investigate trade-offs that adult Belgian hemophilia A and B patients are willing to make when asked to choose between a standard of care and gene therapy; the protocol of which is reported in this manuscript. The survey established through this protocol will allow for exploration of preference heterogeneity and serves to meet the needs of HTA and payers. In the design of the protocol, special attention was given to the innovative nature and potential lack of knowledge of patients regarding gene therapies.

## Aims

The main objectives of the PAVING study are:

- To understand the trade-offs that patients make when they are asked to choose between gene therapy and a standard of care.
- To explore preference heterogeneity by investigating the impact of patient characteristics on preferences.

## Methods and Analysis

### Organization and Patient Involvement

**Figure 1:**
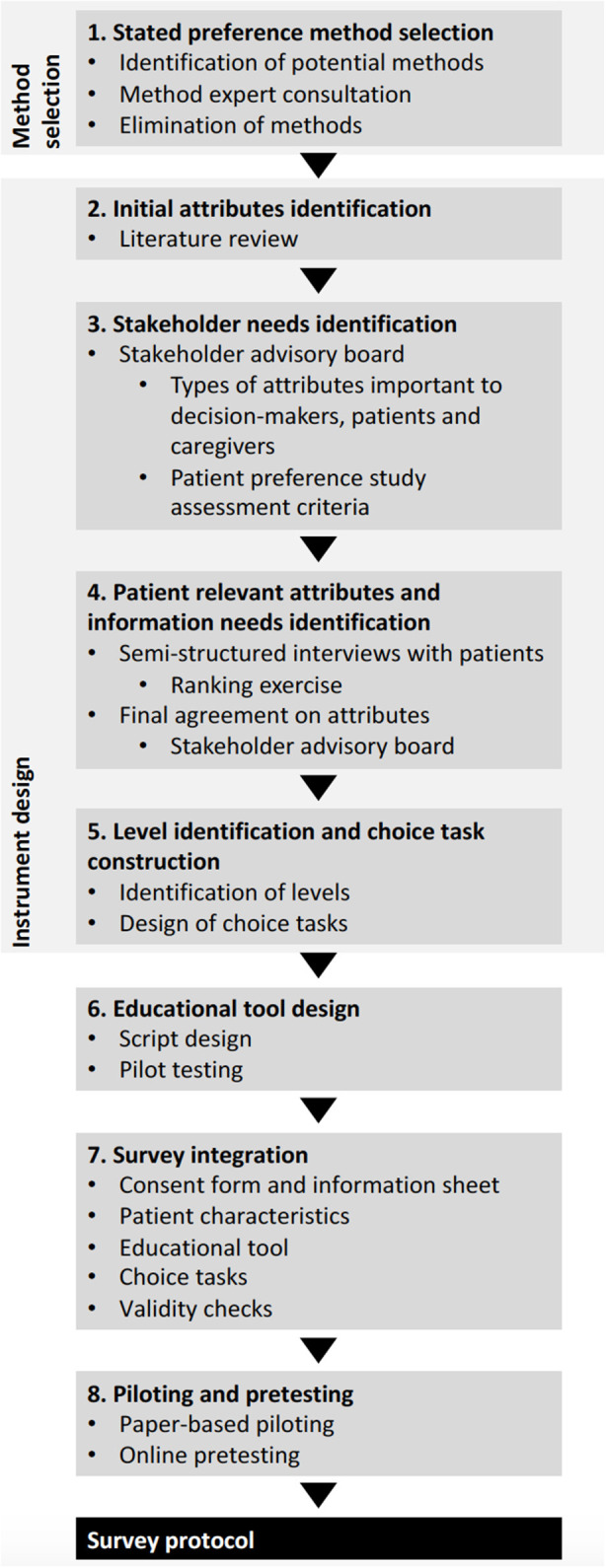
Steps taken in the protocol development.

Protocol development for the PAVING survey was undertaken in sequential steps (Figure 1). Overall, a transparent and systematic approach was taken to develop the protocol, covering steps in the organization, design and conduct of a patient preference study as described by van Overbeeke et al. (14). Patients were involved as advisors (15) in protocol development (steps 3–8), and included in the stakeholder advisory board of the study, that further consisted of hematologists, HTA and payer decision-making experts, industry market access experts, rare disease experts, patient education (EUPATI) experts and caregivers. Moreover, patients steered the selection of attributes through participation in interviews (Step 4).

### Step 1: Stated Preference Method Selection

A number of stated-preference (elicitation) methods exists, but guidance is lacking on when to choose what method. Method selection started from the nine elicitation methods identified by Whichello et al. (16) as most promising in meeting decision-makers' needs in the medical product lifecycle (MPLC): DCE, Threshold Technique, Standard Gamble, Time trade-off, Swing-Weighting, Visual Analog Scale, Analytical Hierarchy Process, Best-Worst Scaling type 1, and Best-Worst Scaling type 2. The match of the method to the research question, patient population and decision-making context influences the value of patient preference studies for decision making (17). Therefore, in selecting our method we used criteria based on the research questions, patient population (rare disease), decision-making context, as well as validity requirements and budget. The criteria used and the thresholds used for this selection were informed by the work of Whichello et al. (16) and discussion with method experts further informed our choice of method. Ideally, we wanted the method to: (1) estimate weights of attributes, (2) estimate trade-offs between attributes, (3) quantify preference heterogeneity, (4) incorporate internal validity measures, (5) not have technical issues, (6) have a low minimal necessary sample size, and (7) allow for incorporation in an unsupervised survey.

While sample sizes of fewer than 100 participants may be sufficient when there is a limited number of attributes and levels (e.g., four attributes each with 2 levels) (18), DCEs typically include more than 100 participants and may require sample sizes >250 if there are 6–8 attributes each with 3–4 levels (16, 19). DCEs were excluded as a method due to our estimation that it will be challenging to recruit 100 patients (see section on sample) (Table 1). Moreover, as described under Steps 4 and 5, we wanted to include four attributes with a maximum of 7 levels in our design. From the nine promising methods, experts initially believed that the threshold technique and swing-weighting showed the most potential to meet study needs. In the end, swing-weighting was excluded based on concerns regarding the need to provide support for participants (i.e., through interviews or workshops) due to complex choice tasks with high cognitive burden, and the threshold technique was chosen.

**Table 1:** Selection criteria applied to the nine preference elicitation methods.

** 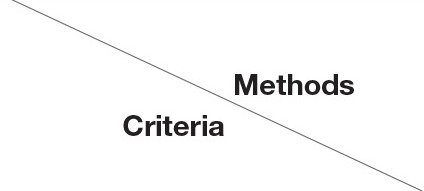 **	**Discrete Choice Experiment**	**Threshold Technique**	**Standard Gamble (20)**	**Time-Trade-Off**	**Swing- Weighting**	**Visual Analog Scale**	**Analytical Hierarchy Process**	**Best-Worst Scaling type 1**	**Best-Worst Scaling type 2**
Estimate weights of attributes	**√**	**√**	**√**	$X^{a}$	**√**	**√**	**√**	**√**	**√**
Estimate trade-offs between attributes	**√**	**√**	$X^{c}$	$X^{c}$	**√**	**√**	$X^{b}$	$X^{b}$	**X**
Quantify preference heterogeneity	**√**	**√**	**√**	**√**	**√**	**√**	**√**	**√**	**√**
Incorporate internal validity measures	**√**	**√**	**√**	**√**	**√**	**√**	**√**	**√**	**√**
Absence of technical issues	**√**	**√**	X	**√**	**√**	**√**	**√**	**√**	**√**
Minimal necessary sample size	>100	20 (21–23)	<100	<100	<10 (24)	<100	<100	<100	>100
Unsupervised survey	**√**	**√**	**√**	**√**	X	**√**	**√**	**√**	**√**

a *Only to investigate time attributes (survival time)*.

b *Not possible to include levels*.

c *Profile-based (not attribute-based) method; can only provide trade-offs if attributes are survival and health state*.

*Red, reason for exclusion*;

*yellow, uncertain/not most optimal choice*;

*green, reason for selection*.

In a threshold technique survey, participants are presented with multiple choice tasks in which they have to choose between two labeled profiles (e.g., prophylactic factor replacement therapy and gene therapy). The level of one attribute in the target profile (gene therapy) is varied systematically until the respondent switches from his/her preferred alternative. The level of this attribute is made systematically better (more attractive) if the reference profile is chosen, or the level of the key attribute is made systematically worse (less attractive) if the target treatment is chosen. The responses to these questions are then used to define an interval per respondent within which their threshold lies. This threshold represents the maximum acceptable risk (MAR) or minimal accepted benefit (MAB) for that switch (25).

### Step 2: Initial Attributes Identification

A literature review was conducted on gene therapy clinical trials and previous initiatives investigating patients' preferences and needs in hemophilia to identify attributes. Clinical trials were identified in PubMed using the search terms “gene therapy” AND “hemophilia” and filters “Clinical Trial” and “Human.” Aditionally, the worldwide clinical trial gene therapy database (26) and clinicaltrials.gov were consulted. Results were cross-checked with the review on hemophilia gene therapy clinical trials of Batty and Pasi (27). Publications reporting results of trials were identified and included if published after 2005 and if intravenous administration of liver-targeting vectors was used. Patient preference studies and public patient meetings were identified in the literature. An initial list of attributes was generated based upon clinical outcomes identified in these clinical trials, and patient relevant outcomes identified in the patient preference studies and public patient meetings.

In total, 18 publications reporting on results from 21 clinical trials were retrieved (Supplementary Material I). Four publications published before 2005 and another publication demonstrating intramuscular application of gene therapy were excluded. In addition, we identified 19 patient preference studies and public patient meetings (Supplementary Material II). Patient preference studies only investigated preferences for treatment attributes of factor replacement therapy, blood transfusion or treatments no longer under development (28). Public meetings of the FDA investigated attitudes of hemophilia patients toward their current therapy and gene therapy (29). From these 13 clinical trials and 19 patient preference studies/public patient meetings, eight attribute classes comprising 22 attributes were identified (Table 2).

**Table 2:** Attributes identified through literature review.

**Classes**	**Attributes**
Nature of treatment	Mechanism of action
Administration	Route of administration
	Dose frequency
	Duration of administration
	Dosage strength
	Place of administration
	Ease of administration
	Ease of product storage
Follow-up	Frequency of monitoring
Benefits	Effect on factor level
	Effect on annual bleeding rate
	Probability that prophylaxis can be stopped after treatment
	Uncertainty regarding long-term benefits
QoL	Impact on daily life
	Impact on participation in physical activity
	Possibility to undergo major surgery
Risks	Probability that liver inflammation will develop
	Uncertainty regarding long-term risks
Costs	Out-of-pocket cost
	Societal cost
Manufacturing	Manufacturer
	Shortage history

*Clinical trials: (30–42)*.

*Patient preference studies/public meetings: (11, 13, 28, 29, 43–57)*.

### Step 3: Stakeholder Needs Identification

To identify classes of attributes important to decision-makers, consultations were held with the advisory board. Attributes identified in Step 2 and value assessment criteria (according to the Belgian Royal Decree of 1 February 2018) were presented and discussed to explore their relevance. Stakeholders confirmed the importance of the presented value assessment criteria and identified the following attribute classes: benefits, risks, administration, level of unmet need, cost and budget impact, applicability, and burden of disease. A consensus among the advisory board was reached on the need to investigate attributes related to benefits (including clinical endpoints and QoL), risks, and administration in the preference study, and to exclude other attribute classes (Supplementary Material III).

### Step 4: Patient Relevant Attributes and Information Needs Identification

To identify attributes to be included in the survey design, relevance of attributes was investigated in interviews with 20 Belgian hemophilia A and B patients. An interview guide for semi-structured interviews with Belgian hemophilia patients was designed. The interview guide was created in Dutch, translated to English and French by a certified translator and checked by one of the researchers (EvO). Patients participated in their native language (Dutch or French). Prior to any questions about gene therapy, patients received information (based on the literature retrieved in Step 2, validated by three hematologists and piloted with two patients) regarding the disease, standard of care and gene therapy (Supplementary Material IV). Overall, patients found the provided information comprehensible. Some patients requested more information on inhibitors against factor replacement therapy, viral vectors, development of light inflammation of the liver, antibodies against vectors, and re-administration of gene therapy if benefits are not maintained in the long-term. Moreover, several patients suggested to use illustrations to visualize difficult concepts and ensure comprehension by other patients.

A ranking exercise was performed during interviews to prioritize attributes according to their importance to patients; using attributes identified through a mixed top-down and bottom-up approach. Top-down attributes included attributes identified in Step 2, except those belonging to classes of attributes excluded in Step 3. Attributes were listed per class and defined (Supplementary Material V). Definitions were validated by three hematologists and pilot tested with two patients. Bottom-up attributes were identified by asking patients to name the top three elements influencing their choice between standard of care and gene therapy before disclosing the top-down attributes. Patients ranked their top six attributes among the top-down and bottom-up identified attributes. This ranking was transformed for each participant so that a score between 1 and 6 was assigned to each of the attributes in the top six, with six points being assigned to the most important attribute. Sum totals of the scores were calculated per attribute. The ranking exercise revealed that the five attributes most important to patients were: annual bleeding rate (ABR), factor level, uncertainty of long-term risks, impact on daily life, and probability that prophylaxis can be stopped (Table 3). Full details on methods and results (on general gene therapy perception) of the interviews have been reported elsewhere (58), according to the guidelines of Hollin et al. (59). In a second consultation with the advisory board the interview results were presented. A consensus was reached to include attributes in the survey that were most important to patients, with emphasis on including a QoL-related attribute.

**Table 3:** Top 10 attributes important to patients.

**Rank**	**Attribute**	Points^*^
1	Effect on annual bleeding rate	47
2	Factor level	43
3	Uncertainty long-term risks	39
4	Impact on daily life	39
5	Probability that prophylaxis can be stopped	32
6	Possibility to undergo major surgery	26
7	Route of administration	21
8	Probability of liver inflammation	21
9	Mechanism of action	20
10	Dose frequency	17

* *n = 18; maximum score is 108 (6 points × 18 interviewees) per attribute*.

To keep the threshold technique survey of manageable length, it was decided to include four attributes. As the meaning of “Factor level” is different for factor replacement therapy (fluctuating factor levels) compared to gene therapy (stable factor levels), and as “Annual bleeding rate” is dependent on “factor level,” the researchers decided to exclude “Factor level” and include “Annual bleeding rate.” “Probability that prophylaxis can be stopped” was rephrased to “Chance to stop prophylaxis” as this was found to be more comprehensible to patients. “Uncertainty regarding long-term risks” was rephrased to “Time that side effects have been studied” as current uncertainty in long-term risks of gene therapy is caused by limited follow-up in a relative small number of patients (60); a similar attribute has been used by Mohamed et al. (61). In addition, a “Quality of life” attribute similar to Tomlinson et al. (62) was chosen as a substitute for “Impact on daily life,” as no hemophilia-specific impact on daily life instrument exists. The final selection of attributes thus included three benefits: “Annual bleeding rate” (ABR), “Chance to stop prophylaxis” (STOP) and “Quality of Life” (QOL); and one risk: “Time that side effects have been studied” (TIME). Attributes were further defined, and definitions were validated by three hematologists.

### Step 5: Level Identification and Choice Task Construction

**Figure 2:**
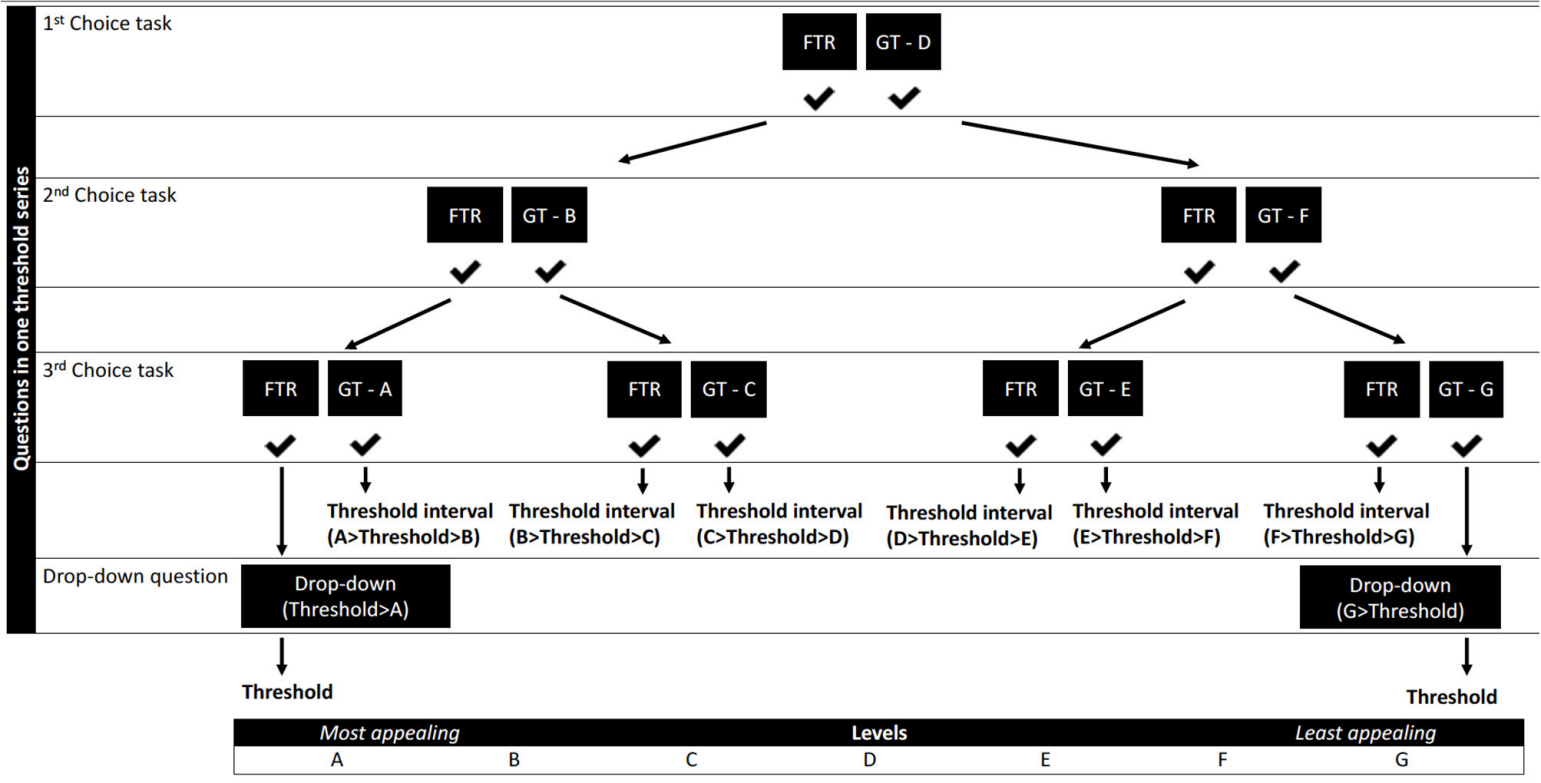
Flow of the levels throughout the questions of one threshold technique series. GT - A-G, gene therapy levels A-G (Table 4); FTR, factor replacement therapy level.

Three threshold series comprising up to three choice tasks and a drop-down question were designed to identify threshold intervals, one for each benefit (“Annual bleeding rate,” “Chance to stop prophylaxis” and “Quality of Life”). We opted to ask up to three choice questions per threshold series to each individual participant as shown in Figure 2 as this is often the number of questions used in threshold technique surveys to identify individual thresholds. As demonstrated in Figure 2, seven levels (levels A-G) were required to complete the design. Attribute levels were identified through literature gathered in Step 2, hematologist consultation and additional literature on QoL scores in hemophilia patients (63–67).

The range of attribute levels was based on the best available clinical data at the time this protocol was designed. From the 18 publications identified in Step 2 that reported on results from Phase I/II gene therapy trials in hemophilia (Supplementary Material I), one publication (68) was excluded as it described an intramuscular application of gene therapy and four other publications were withheld as they were published before 2005 and therefor found to be outdated (69–72). From the remaining 13 publications, lower and upper bounds of levels were identified and a range was set for all attributes using the lowest and highest value identified across publications (30–42). As QoL was not yet studied in these trials, we hypothesized that gene therapy would at least not reduce QoL and current QoL levels were identified using five additional studies (63–67). The ranges of the levels were discussed with hematologists (*n* = 3) and the range of the TIME attribute was slightly adapted based on their input to reflect the number of years of available evidence at that time (Table 4).

**Table 4:** Levels for the threshold technique survey.

**Attributes**		**Levels**								
		**FTR (63–67)**	**Gene therapy**							
			**Ranges (30–42)**	**Levels used in the survey**						
				**A**	**B**	**C**	**D**	**E**	**F**	**G**
Benefits	Annual bleeding rate	6^*^	0–11	1	3	5	6	7	9	11
	Chance to stop prophylaxis (%)	0^*^	33.3–100		100	95	90	85	80	75
Uncertainty	Time that side effects have been studied years	30^*^	3–10^*^				10			
Quality of life	Quality of life	70	0–100^**^	85	80	75	70	65	60	55

* *Adapted based on discussion with hematologists*.

** *Unknown; the minimum and maximum levels of the attribute scale are presented*.

*FTR, factor replacement therapy (prophylactic)*.

A threshold technique response logic was created using levels within the identified ranges (Table 4 and Figure 2). Spacing of these levels was established by setting the most extreme values of these ranges as cut-offs. We aimed to obtain even spacing between levels, with a maximum spacing of five units between levels. The threshold technique requires one attribute to be fixed as a comparator (25). It was decided to keep the risk attribute “Time that side effects have been studied” constant at level D throughout the threshold questions, to enable estimation of patient preferences for all benefit attributes. Levels D of all attributes represent the gene therapy profile in the initial threshold question. These levels represent the baseline scenario on which all threshold estimates are contingent. The levels for PFRT and the gene therapy baseline scenario were also fixed in discussion with hematologists to reflect a conservative scenario, where gene therapy would not provide additional ABR and QoL benefits, that still fit within the identified level ranges (Table 4).

The levels of only one benefit will change throughout a threshold series to identify an individual's threshold for that benefit. With a total of three series and with the initial question (levels D) representing the first choice task of all threshold series, participants need to answer seven choice questions in total to obtain a threshold interval for the three benefits within which their individual thresholds will lie. If participants end up at the extreme ends of Figure 2, no threshold interval can be identified and participants will be asked an additional drop-down question to elicit their exact threshold.

### Step 6: Educational Tool Design

To ensure comprehension of the attributes and the gene therapy context by participants, an educational tool was designed. The information presented in the educational tool comprised hemophilia, current therapies and gene therapy and covered information needs of patients as identified in Step 4 (Supplementary Material VI). The original English script was translated to Dutch and French translations by a researcher (EvO) and validated by a certified translator. Voice-overs were recorded and Mindbytes BVBA developed the educational tool with visuals according to their standards (73).

The content and visuals of the educational tool were reviewed by three hematologists, two patients and a patient education expert. Necessary changes to the tool were made, and the Dutch and French versions were piloted with 10 additional patients. Patients were asked how comprehensible the tool was to them (user comprehensibility) and how comprehensible it would be to other patients. User comprehensibility of all modules was rated between “Very comprehensible” and “Totally comprehensible;” except the side effects module that was rated as “Comprehensible” by one patient. Comprehensibility to other patients was rated between “Comprehensible” and “Very comprehensible” across all modules. Ease of navigation was rated by all patients between “Very easy” and “Easy”. In addition, six patients reported that no changes needed to be made to the educational tool, two mentioned minor navigation changes and two requested additional information (on antibodies and gene therapy re-administration). Overall, the tool was very well-received by patients. Therefore, no additional changes were made.

### Step 7: Survey Integration

The final survey was designed to include (1) a consent form and information sheet, (2) questions on patient characteristics including demographics, health literacy Chew et al. (74) and QoL (EQ5D5L), (3) the educational tool established in Step 6, (4) the choice tasks using the threshold technique as designed in Step 5, and (5) survey evaluation questions. Questions on demographics (e.g., age, disease severity, number of damaged joints) and on QoL (EQ5D5L) were included to identify factors that may influence preferences of patients. Health literacy questions were included to identify patients that may have difficulties with understanding medical information. To evaluate the validity of the study, validity checks were built into the survey to identify respondents whose responses appear to “fail” these validity checks based on expected norms. Validity checks included evaluation of a comprehension question similar to that of Mansfield et al. (75), time to complete the survey, and choice consistency (the initial threshold question was repeated after the first threshold series). Dutch and French translations of the English survey were made by a certified translator and reviewed by a researcher (EvO), excluding QoL questions for which validated translations were used. The survey was programmed by Qualtrics and thoroughly reviewed by the researchers.

### Step 8: Piloting and Pretesting

The full survey was piloted and pretested with patients. Four patients (including two bilingual patient representatives) participated in a paper-based pilot that evaluated comprehensibility of Dutch and French choice questions and choice behavior in think aloud interviews (76, 77). During this pilot no major issues were found and only minor text edits were made to a definition of one attribute and one question.

Online unsupervised pretesting evaluated comprehensibility and length of the survey, functioning of the response logic, and ability to identify thresholds and trade-offs. Of 14 invited patients, 12 completed the online pretest. The majority of pretesting participants found the choice questions to be “Very easy” or “Easy” to understand and answer. Some found it “Not easy nor difficult,” and none found it “Difficult” or “Very difficult”. Participants found the survey length “Just right” (*n* = 3), “Manageable” (*n* = 7), or “Too long” (*n* = 2). However, seven participants took over 40 min to complete the survey. Two of these participants had paused the survey and others might have taken a longer time than expected as they were also asked to evaluate the survey. Participants reported no other issues besides one textual error in the consent form and two in demographics questions. Therefore, the textual errors were corrected and three demographics questions were excluded to reduce the length of the survey. Inspection of the data sheet confirmed correct functioning of the response logic and ability to identify thresholds and trade-offs. The final survey can be found in Supplemental Material VII.

### Sampling and Recruitment

No specific power calculation method exists to determine sample sizes for threshold technique studies. Most threshold technique studies are conducted with 100 or fewer respondents (successful small studies include between 20 and 42 respondents) (18, 21–23). The threshold technique allows for elicitation of individual preferences (*n* = 1) and the method can therefore be used in very low sample sizes. The significance of the estimates will be greater and standard deviations will be smaller when the sample size increases. Hemophilia is a rare disease but relatively common compared to other rare diseases. The number of people affected by hemophilia A and B in Belgium was 1 258 in 2018 (78). Based on this number we estimate that we will be able to include around 100 patients in Belgium, and a method expert confirmed that the method can be performed with this limited proposed sample size.

Patients will be considered eligible if they are diagnosed with moderate or severe hemophilia A or B, are 18 years or older, and live in Belgium. Patients will be recruited through national hemophilia reference centers and the national patient organization. These recruiting parties will send an invitation via mail or newsletters containing a link to the online survey. Recruiting parties will keep a record of the number of eligible patients they sent an invitation to so that response rates can be calculated.

### Analytical Plan

Analysis of thresholds and trade-offs will be done through interval regression and plotting of thresholds. Threshold intervals will be analyzed per benefit attribute (ABR, STOP, and QOL) using two interval regression (Tobit) models: (1) a constant-only model to identify the mean threshold (MAB) across the sample, and (2) a covariate-adjusted model to explore whether and how patient characteristics influence the MAB for each benefit (i.e., to explore preference heterogeneity). A separate Tobit model will thus be run for each benefit attribute.

A number of patient characteristics will be tested for inclusion in the covariate-adjusted model which may explain some of the observed preference heterogeneity. These include sociodemographic characteristics (e.g., age, residence, employment status), medical characteristics (e.g., hemophilia type, disease severity, and self-reported ABR and QoL), and survey behavior characteristics (e.g., time spent on the educational tool). The final selection of patient characteristics to be included in the covariate-adjusted model will be based on results from correlation tests between these covariates.

## Discussion

This research resulted in the development of the PAVING protocol to investigate trade-offs that adult Belgian hemophilia A and B patients are willing to make between standard of care and gene therapy. To the authors knowledge, this is the first patient preference study protocol that has been designed in the context of market access of gene therapies.

A transparent and systematic approach was taken to develop the PAVING protocol. While protocols of preference studies explaining the choice of attributes are increasingly being published (79–82), it is not standard practice to justify the choice of the preference method and the choice is often DCE (83). However, depending on the research question, researchers may prefer other methods over DCEs in case of very small sample sizes. The current research resulted in a transparent selection of a preference method (i.e., the threshold technique), attributes and levels. The protocol adheres to the five considerations of van Overbeeke et al. (17) to ensure value of a preference study for decision making: (1) investigate preferred treatment attributes, and trade-offs between attributes, (2) have a design that matches the research question and patient population, (3) include a patient sample and method that matches the MPLC phase, (4) be conducted in collaboration with different stakeholders, and (5) allow for sharing of results with relevant stakeholders.

The researchers believe that by taking a patient-centered approach (i.e., involving patient throughout protocol development and conducting interviews with patients) attributes were included that are relevant and comprehensible to patients (15). The research resulted in the inclusion of the attributes “Annual bleeding rate,” “Chance to stop prophylaxis,” “Quality of Life,” and “Time that side effects have been studied”. While “Quality of Life” may not be a usual attribute to include in a preference study, our QoL attribute is reliable as it will visually be presented as the EQ5D visual analog scale (VAS) that ranges from 0 (worst possible QoL) to 100 (best possible QoL). The researchers also believe that this VAS scale (reflecting patients' own valuation of their health) is easier to understand to patients and that results using this scale are easier to interpret than when using the utility scale that goes from 0 (death) to 1 (full health), as these utilities can go below 0 and the scale reflects a societal valuation of health states. Moreover, the QoL attribute is described according to the five dimensions of EQ5D5L [a reliable tool to measure QoL (84)] to make the attribute concrete. Potential concerns regarding ambiguity of QoL reflect the limitations of its current use as a generic measure of value in decision-making. While QoL may not be fully independent from ABR, bleedings do not occur on a daily basis, and patients can have different QoLs with the same ABR and also have the same QoL with different ABRs; to the extent of realistic ABR and QoL levels. As the threshold technique allows for the use of realistic levels within labeled profiles, the researchers argue that QoL and ABR can both be included as attributes. In contrast, simultaneous use of these two attributes in a DCE may not be possible as hypothetical scenarios may for example present unrealistic high ABR in combination with high QoL, possibly leading to rejection by patients. As demographics and QoL of patients will be investigated, clinical independence between the two variables, and the relation between current QoL and preferences can be investigated.

An important limitation of our design is that interactions between attributes cannot be assessed. Potential effects of uncertainty in risks (time that side effect have been studied) on interpretation of benefits can thus not be studied. Anchoring effects are always a possible limitation in any survey in which one value is changed systematically until switching or indifference is achieved. This is true for time tradeoff and standard gamble, modified swing weighting, and the threshold technique. However, to the extent that the baseline level to which the decision is anchored represents reality “in that it is based on data or on a value that would be expected even if data do not exist, then the starting point reflects the true decision context and will reflect bias inherent in that decision context” (25). In our case, the levels of each attribute in the initial (i.e., baseline) question, represent levels likely to be associated with the relevant alternatives (factor replacement and gene therapy) according to the clinical evidence available at time protocol design, and therefor may reflect a real-world decision context. However, as Phase III trial data still has to become available and uncertainties about the outcomes of gene therapy in hemophilia exist, the relevance of the baseline scenario may be affected by new clinical data becoming available. Therefore, the results of this study should always be interpreted relative to the latest available clinical data (85).

Comprehension of the survey by participants will be ensured through use of the educational tool that was designed. Vass et al. (86) showed in their study that the use of an animated educational tool did not change preferences of respondents, but improved choice consistency. The information presented in the educational tool developed in the current research covers information needs of patients and was validated by hematologists and piloted with patients. Moreover, the tool also covers different aspects highlighted in the work of Barber et al. (60), including but not limited to uncertainty in long-term safety and efficacy, eligibility criteria, variability in achieved outcomes, and current absence of major safety issues.

It should be acknowledged that the data presented in this paper, and that informed protocol development, was elicited from a small sample of stakeholders and patients. While this approach is appropriate for development of stated preference protocols and is supported by an extensive literature review, a larger sample would be required to reach representativeness of results.

## Ethics and Dissemination

Ethics approval was sought and granted by the Medical Ethics Committee of UZ KU Leuven/Research in Belgium for both the interviews (S62670) that informed this protocol, as well as the conduct of the PAVING survey (S63686). In addition, the ethics committee also approved the analysis plan and data management plan of the study. Prior to the interviews, all interviewees provided written informed consent. Survey participants will be informed that their participation is anonymous and that, to ensure anonymity, they will not be able to view, edit or remove responses once submitted. They will then be asked to provide electronic informed consent before they can answer any questions in the survey. An open text question included at the end of the survey will allow participants to raise any concerns.

Results of the study will be communicated to stakeholders through publications. Results will also be disseminated at clinical and health economic conferences, and will be presented to the advisory board of the study. Moreover, the researchers plan to write a lay language summary of the results to be distributed to patients via the recruiting parties.

Learnings gained through the development of this protocol and the results of the PAVING study may:

- Inform Belgian HTA and payer (and potentially also regulatory) decision-making on gene therapies, by providing insights on the elements of these therapies that patients value, and the acceptability of long-term safety uncertainties. Moreover, the results from the PAVING survey can demonstrate what gene therapy profiles will be acceptable to patients, while also showing the potential existence of preference heterogeneity.
- Lead to the design of similar studies in hemophilia to inform decision making in other countries. While this protocol was setup to specifically meet needs of the Belgian market access setting, the included attributes may also be relevant for HTA/payers in other countries. Before this protocol can be used in other countries, it should be investigated if HTA representatives and payers in other countries believe that attribute classes excluded in this study should be explored in a preference study. Moreover, we advise researchers interested in using this protocol in another country, to perform interviews with patients similar to our interviews to confirm whether the selected attributes are also important to patients in their country of interest.
- Inspire other researchers to conduct similar gene therapy patient preference studies in different disease areas. This protocol describes how the unique features of gene therapies can be transformed to attributes and included in preference studies. While some of the attributes described in this protocol are specific to hemophilia, the researchers would like to encourage others to apply the PAVING approach and use similar attributes in other disease areas where gene therapies are in development.

## Ethics Statement

The studies involving human participants were reviewed and approved by Medical Ethics Committee of UZ KU Leuven/Research. The patients/participants provided their written informed consent to participate in this study.

## Author Contributions

EO, BH, SM, MG, SS, and IH were involved in the development of the protocol. In Step 1–3, EO performed literature reviews, held meetings with methods experts and the advisory board to inform method and attribute selection. In Step 4, EO and SM designed study materials, conducted interviews with patients, and analyzed them. In Step 5, EO designed the choice tasks and response logic. In Step 6, EO and SM designed the educational tool in collaboration with Mindbytes BVBA. In Step 7 and 8, EO was responsible for the integration of the survey, coordination with Qualtrics, and conduct of the pilot and pretest. Along all steps BH, MG, SS, and IH participated in meetings and reviewed materials. EO produced the first draft of the manuscript, which was subsequently revised and finalized with all authors. All authors contributed to the article and approved the submitted version.

## Conflict of Interest

The authors declare that the research was conducted in the absence of any commercial or financial relationships that could be construed as a potential conflict of interest.
