# Supplementary material for: Patient Preferences to Assess Value IN Gene Therapies: Protocol Development for the PAVING Study in Hemophilia
Source: Front Med (Lausanne). 2021 Mar 9;8:595797. doi: 10.3389/fmed.2021.595797 (PMC7985056; doi:10.3389/fmed.2021.595797)
Supplement: Supplementary file 1 [file Data_Sheet_1.PDF]

Supplementary material - Patient preferences to Assess Value IN Gene therapies: protocol development for the PAVING study in hemophilia

van Overbeeke E, Hauber B, Michelsen S, Goldman M, Simoens S, Huys I

Table of Contents

Supplementary material I Identification of gene therapy clinical trials in hemophilia 2

Supplementary material II Identification of patient preference studies and public patient meetings in hemophilia 3

Supplementary material III Identification of stakeholder needs 6

Supplementary material IV Information provided to patients in interviews...................... 7

Supplementary material V Ranking exercise performed with patients in interviews 9

Supplementary material VI Script of the educational tool 10

Supplementary material VII Final survey (English) 12

References...57

Supplementary material I Identification of gene therapy clinical trials in hemophilia

Date of search: 8th of December 2018
Search engine: PubMed
Search terms: “gene therapy” AND “hemophilia”
Filters: “Clinical Trial” and “Human”
Additional sources: Worldwide Clinical trial gene therapy database (1), clinicaltrials.gov, and the review on gene therapy clinical trials of Batty and Pasi (2).

Table III.I Gene therapy clinical trials in haemophilia

Vector (sponsor)	*Date initiated*	*Status*	*Number of patients included*	*Doses (vg/kg)*	*Mean outcome (factor level; % of normal)*	*Phase*	*Trial number (results reference)*
Haemophilia B (Factor IX)							
scAAV2/8-LP1-hFIXco (UCL and SJCRH)	2009	Closed	2<br>2<br>6	2x10^{11}<br>6x10^{11}<br>2x10^{12}	1.8 (1.4-2.2)<br>2.5 (2.2-2.9)<br>5.1 (1.4-7.2)	Phase I/II	(3, 4)<br>NCT00979238
SPK-9001 (Spark Therapeutics)	2009	Closed	10	5×10^{11}	33.7 (14-81)	Phase I/II	(5, 6)<br>NCT00979238
Fidanacogene elaparvovec (SPK-9001; Pfizer)	2018	Open	NR	NR	NR	Phase III	NCT03587116 (lead-in study)<br>US-1742
AMT-060 (UniQure)	2015	Closed	5<br>5	5x10^{12}<br>2x10^{13}	4.4 (1.3-6.8)<br>6.9 (3.1-12.7)	Phase I/II	(7, 8)<br>NCT02396342<br>EudraCT#2013-005579-42
AMT-061 (UniQure)	2018	Open	NR	2x10^{13}	NR	Phase III	NCT03569891
FLT180a (Freeline Therapeutics)	2018	Open	NR	4.5x10^{11}	NR	Phase I/II	NCT03641703<br>(9)
SB-FIX (Sangamo)	2016	Open	NR	NR	NR	Phase I/II	NCT02695160
AskBio009 (Shire)	2012	Closed	NR	NR	NR	Phase I/II	NCT01687608
DTX201 (Bayer)	2018	Open	NR	NR	NR	Phase I/II	NCT03588299
Coagulin-B (Avigen)	1999		3	2x1011	>1	Phase I	(10)
The Children’s Hospital of Philadelphia	2006	Closed	7	2x10^{12}	NR	Phase I/II	(11)
The Children’s Hospital of Philadelphia	2006	Closed	8	2x10^{11}<br>1.8x10^{12}	<1	Phase I	(12)
Fudan University	1993	Closed	2	NR	220 ng/ml	Phase I	(13)
Haemophilia A (Factor VIII)							
Valoctocogene roxaparvovec (BMN-270; BioMarin)	2015	Closed	6<br>7	4x10^{13}<br>6x10^{13}	51 (48-54)<br>86 (65-107)	Phase I/II	(14-16)
Valoctocogene roxaparvovec (BMN-270; BioMarin)	2018<br>2017	Open<br>Open	NR	4x10^{13}<br>6x10^{13}	NR	Phase III	NCT03392974<br>NCT03370913<br>US-1676<br>US-1691
SPK-8011 (Spark Therapeutics)	2016	Open	2<br>3<br>7	5×10^{11}<br>1x10^{12}<br>2x10^{12}	13<br>15<br>30 (16-49)	Phase I/II	NCT03003533<br>(17)
SB-525 (Sangamo)	2017	Open	NR	NR	NR	Phase I/II	NCT03061201
SHP654 (Shire)	2017	Open	NR	NR	NR	Phase I/II	NCT03370172
GO-8 (UCL)	2016	Open	NR	6x10^{11}<br>2x10^{12}	NR	Phase I	NCT03001830<br>(18)
hFVIII(V) (Chiron, Emeryville, CA)	2003	Closed	13	2.8x10^{7}<br>- 8.8x10^{8}<br>TU/kg	>1	Phase I	(19)
Non-viral (Transkaryotic Therapies)	2001	Closed	6	NR	<0.8	Phase I	(20)

NR, Not Reported. UCL, University College London. SJCRH, St. Jude Children’s Research Hospital.

Supplementary material II Identification of patient preference studies and public patient meetings in hemophilia

**Table IV.I** Patient preference studies and public patient meetings in hemophilia					
Study	*Year*	*Aim*	*Method*	*Treatments*	*Outcomes*
FDA workshop (21)	2016	To hear experience with bleeding disorders and their treatment	Public meeting	Current + future options	The ideal treatment would be a cure, through gene therapy or a transplant, with no accompanying side effects and no need for continuing maintenance of therapy. Patients want safe, effective, potent, inexpensive, very long acting, and easy to administer products. Patients are willing to take part in a trial only for "better option – a curative gene therapy or a treatment with a longer half- life."
FDA workshop (21)	2018	To understand patient/caregiver concerns, perceived risks and benefits, and expectations of gene therapy	Public meeting	Gene therapy	Accurate information on gene therapy if important. Patient/caregiver's interest in new therapy is partly dependent on the success of their current treatment modality; uncertainty relating to benefits and risks influences acceptance. Benefit: no further infusions, ability to be more active, prevention of additional pain and joint deterioration. Frequency of safety monitoring was not seen as a high priority. Some patients/ caregivers would be satisfied with Factor IX levels of 5-10% while others indicated they hope for levels of 40% or more. Some patients/ caregivers would be satisfied with a duration of elevated Factor levels for 5 years, while others indicated they hope for a duration of more than 10 years.
Tael et al. (22)	2014	To develop a brief, clinically relevant tool to measure ease of use and patients' preference for haemophilia treatment	40-item questionnaire	Factor replacement therapy	The study resulted in the setup of a 14-item questionnaire - the HaemoPREF (tested in Bonanad et al. (23))
Bonanad et al. (23)	2017	Patient perception of Haemophilia A treatment	HaemoPREF	Factor replacement therapy	Important attributes: dosing frequency, ease of use, risk (viral infections and inhibitor development), and impact on daily life.
Chaugule et al. (24)	2015	Preferences and willingness to pay (WTP)	Discrete choice experiment	On-demand and long-acting factor replacement therapy	Out-of-pocket treatment costs, side effects, and treatment effectiveness and dosing frequency were found to be statistically significant in the model

**Table IV.I (continued)** Patient preference studies and public patient meetings in haemophilia (25)					
Study	*Year*	*Aim*	*Method*	*Treatments*	*Outcomes*
Brown et al. (26)	2011	Identify treatment attributes that patients/caregivers consider most important	Discrete choice experiment	Factor replacement therapy	The three most important attributes: time required to stop bleeding, possibility that the level of inhibitor may rise and risk of contracting a virus from the product
Steen Carlsson et al. (27)	2017	Valuation of treatment attributes	Time Trade-Off	Factor replacement therapy	Important attributes: Participation in physical activity, duration of injection interval; frequency of bleeds
Furlan et al. (28)	2015	To identify the relative importance of frequency of administration, efficacy, and other treatment characteristics	Conjoint analysis	Factor replacement therapy	Important attributes: administration, efficacy, and manufacturer
Mantovani et al. (29)	2005	To identify treatment preferences	Discrete Choice Experiment	Factor replacement therapy	Important attribute: Distribution from home or community pharmacist instead of hospital
Mohamed et al. (30)	2011	To quantify patient and parent preferences	Conjoint analysis	Factor replacement therapy	Attributes in decreasing order of importance: Risk of viral infection, developing inhibitor risk, dosage strengths, percent of bleeds stopped, shortage history, preparation volume.
Scalone et al. (31)	2009	To evaluate preferences towards characteristics of coagulation factor concentrates for haemophilia inhibitors patients	Discrete Choice Experiment	Factor replacement therapy	Important attribute: Allowment to undergo major surgery
DiBenedetti et al. (32)	2014	To assess preferences with FVIII product storage and stability	Survey	Factor replacement therapy	Important attribute: Ease of product storage (longer storage on room temperature)
Costea et al. (33)	2009	Explore patient perspectives	Survey	Genenetically modified autologous adult stem cells	Acceptable symptom free time interval would be 1 month till 1 year. Short term and long term safety is important.
Lock et al. (34)	2016	To evaluate barriers and facilitators for individualized pharmacokinetic (PK)-guided dosing	Discrete Choice Experiment	Factor replacement therapy	If bleeding was consequently reduced, more frequent infusions were acceptable. However, daily dosing remained an important barrier for all involved. 'Reduction of costs for society' was a facilitator for implementation in all groups.
Cimino et al. (35)	2014	To identify preferences for different administration systems	Survey	Factor replacement therapy	Ease of use: the device scenario requiring the least equipment and reconstitution steps (the DCS) received the highest preference rating

**Table IV.I (continued)** Patient preference studies and public patient meetings in haemophilia (25)					
Study	*Year*	*Aim*	*Method*	*Treatments*	*Outcomes*
Musso et al. (36)	2010	To identify preferences for different administration systems	Survey	Factor replacement therapy	The ease of use, perceived safety from needlesticks, and the speed of reconstitution were identified as main advantages by the majority of patients.
Moia et al. (37)	2013	To elicit patient preferences, and estimate their willingness to pay for the different treatment options	Discrete Choice Experiment	Across treatments	Important attributes: route and number of medication administrations, frequency of monitoring, risk of some minor bleeding, and out-of-pocket treatment cost.
Wasserman et al. (38)	2005	To develop a disease-specific utility instrument that measures patient preferences for various health states unique to hemophilia	Visual analog scale (VAS) and standard gamble (SG)	Health states	Adult participants took more risk than paediatric. SG yielded higher preference scores than the VAS for the majority of health states.
Barlow et al. (39)	2007	Burden of disease	Semi-structured interviews	Blood transfusions	Majore impact of infected transfusions on QoL + prefer treatment by specialist over GP

Supplementary material III Identification of stakeholder needs

The first consultations with the advisory board resulted in the identification of classes of attribute important to decision-makers (Table X) and the following assessment criteria for the study:

1. Representativeness: although less important as the study is conducted in a rare disease
2. Independence: the study should be conducted by a neutral party and not by one company
3. Methodology: a mixed-methods approach would be best
4. Patient involvement: to ensure patient understanding and relevance in design and conduct, and ensure correct interpretation of results
5. Attribute phrasing: prevent subjective/steering formulation and provide definitions of attributes
6. Level selection: levels needs to be as realistic as possible and the range of levels should be defined based on clinical results
7. Reliability: validity tests have to be included to demonstrate reliability.

One member of the advisory board also mentioned that it has to be made clear how the patient preference study can relate to costs and cost-effectiveness while not including costs as an attribute when the study would be submitted to HTA bodies/payers.

Table V.I Classes of attributes important to decision-makers

Classes	Decision on use as treatment attribute	Reason for exclusion
Benefits<br>(clinical endpoints and Quality of Life)	Include	NA
Risks	Include	NA
Administration	Include	NA
Level of unmet need	Exclude	Level of unmet need might influence preferences but is not a treatment attribute. Level of unmet need should be captured in demographics/clinical characteristics of participants.
Cost and budget impact	Exclude	Gene therapy prices are not known yet, and the cost and budget impact will be evaluated in HTA/reimbursement decisions separately.
Applicability	Exclude	The results from patient preference studies could be used to inform applicability (The extent to which the drug characteristics, e.g. contraindications, limit the drug use for certain groups of patients and/or require special precautions).
Burden of disease (for patients, family and society)	Exclude	Burden of disease might influence preferences but is not a treatment attribute. Burden of disease should be captured in demographics/clinical characteristics of participants to investigate correlation between burden of disease and preferences.
Abbreviations: NA, not applicable		

Supplementary material IV Information provided to patients in interviews

I will go through some information with you on the disease, current treatment options and the use of gene therapy in haemophilia. After every information section, I will ask you if the information is understandable and how I can improve the phrasing of the information to make it clearer. You might already know about some of the aspects that I will inform you about, but I still would like to receive your feedback on these information sections.

<u>Information on haemophilia</u>
Patients with haemophilia have an error in the gene for a certain coagulation factor, or for short referred to as factor. The error is located in the gene for coagulation factor IX in haemophilia B and the gene for coagulation factor VIII in haemophilia A. Because of this error, these patients are not able to produce the coagulation factor or they produce insufficient amounts of correct coagulation factors. Due to this insufficiency these patients bleed for a longer time compared to people with the correct gene, or bleedings can occur spontaneously in patients that produce almost no correct coagulation factor.

- How understandable was this information to you?
    - How can this information be formulated more clearly?

<u>Information on the treatment of haemophilia</u>
The aim of treatment in haemophilia is to supplement the body with coagulation factor to stop or prevent bleedings. Coagulation factor cannot be swallowed in pill form but has to be injected directly into a vein to reach the blood circulation. Patients can self-administer the coagulation factor when a bleeding occurs or when they know that they will participate in an activity with the risk of causing a bleeding, this is called treatment 'on demand'. In addition, patients can also treat themselves in a 'prophylactic' manner through self-administration multiple times per week to keep the coagulation factor up to standard. For haemophilia A patients this includes two to three administrations per week and for haemophilia B patients two per week. However, the number of necessary injections per week can vary per individual. Some patients develop neutralising antibodies (inhibitors) against the administered factor, resulting in inefficacy of the treatment.

- How understandable was this information to you?
    - How can this information be formulated more clearly?

<u>General information on gene therapy in haemophilia</u>
Genetic disorders are caused by an error that is present in our genetic material, in other words an error in one of our genes. This error can arise spontaneously or can be passed along by one or both parents. By means of gene therapy we try to correct the error so that the body contains the correct gene and the correct activity can take place in the body. The goal of gene therapy in haemophilia is to deliver the correct gene of the coagulation factor to the body. Hereby the correct factor will be produced in the body, and the patient no longer has to administer extra factor via injection.

- How understandable was this information to you?
    - How can this information be formulated more clearly?

With gene therapy the correct version of the gene for the coagulation factor will be administered directly to the body. The correct gene will be packaged in "a vector" that is responsible for delivering the gene to the liver cells, where the new gene is added next to the genetic material that is already present. It will not alter your own genetic material. For haemophilia, a modified virus is used as vector as this has a good capacity to reach the liver cells. The virus is modified in a way that it is only capable of delivering the genes to the liver cells, the virus itself is not infectious or functional. Only the casing of the virus remains, as a sort of taxi.

- How understandable was this information to you?
    - How can this information be formulated more clearly?

<u>Practical information on the treatment of haemophilia with gene therapy</u>
Gene therapy is administered once via a vein in a hospital during 30-60 minutes on average. After the vector has delivered the correct gene to the liver cells, the liver cells themselves start to produce coagulation factor. After this one-time administration the patient will have to come to the hospital for

check-ups regularly during a period of about 3 months to monitor the factor level. After these 3 months this becomes a yearly check-up. The treatment with gene therapy results in a factor concentration that is always on the same level. This in contrast to injections with coagulation factor that results in a high factor concentration directly after injection, but a low concentration before the next injection. This means that gene therapy will provide a stable factor level that is high enough to protect you against bleedings, against fluctuating factor levels with the factor injections.

- How understandable was this information to you?
    - How can this information be formulated more clearly?

Side effects of gene therapy in haemophilia

In some patients a light inflammation of the liver is observed after treatment with gene therapy. This inflammation cannot cause hepatitis C and does not cause noticeable symptoms. The light inflammation will be treated with cortisone (corticosteroids) to avoid the occurrence of symptoms. In addition, 30-50% of the population already has antibodies against the used vectors. This means that these people currently do not qualify for treatment with gene therapy as the vector will be broken down by their body. Because of the presence of these antibodies the vectors will not reach the liver cells and the gene therapy will not be effective. When patients without pre-existing antibodies participate in clinical trials, it has been determined that they always develop antibodies against the vector after administration of gene therapy, this is a normal reaction of the body. This development of antibodies does not cause noticeable symptoms and does not hinder the function of the administered gene therapy. This means that they can be treated successfully with the same vector once, but that treatment with the same vector cannot be repeated. It is unknown if it is possible to treat patients again with another type of vector for which the patient has not yet developed antibodies, if the gene therapy would not work long enough.

- How understandable was this information to you?
    - How can this information be formulated more clearly?

Efficacy of gene therapy in haemophilia

This therapy is still in clinical development and is not yet approved by the European Commission. All data that we present here originate from clinical trials with severe haemophilia patients. Currently, only adult patients with severe haemophilia and without inhibitors can receive gene therapy. In these studies, we see that there is a large variability across results. Some patients no longer need factor injections after gene therapy was administered to them and experience no to almost no bleedings anymore. In contrast, other patients still need extra factor administration and experience a few bleedings per year. To date, no patients have developed inhibitors against the coagulation factor produced by the liver after administration of gene therapy. The monitoring of patients in clinical trials is now 2 years on average for haemophilia A and 8 years for haemophilia B, whereby it is uncertain for how long this therapy will results in sufficient production of coagulation factor by the liver. In other words, it is unknown whether the therapy will provide a life-long effect. In addition, it is expected that gene therapy for haemophilia will come with a one-time high cost for the government against the spread cost that is currently paid for lifelong factor administrations.

- How understandable was this information to you?
    - How can this information be formulated more clearly?

Supplementary material V Ranking exercise performed with patients in interviews

In the table below, please indicate the six elements that you think are most important when deciding whether gene therapy is the right treatment for you/your child (using scores from 1 to 6, with "1" being the most important element).

Categories	***Elements***	*Definition*	***Ranking***
Nature of treatment	**Mechanism of action**	The specific process through which a treatment produces its effect (e.g. through delivering a gene to liver cells in the case of gene therapy; or through delivering factor to the body in the case of factor replacement therapy)	
Administration	**Route of administration**	The path by which a treatment is administered to the body (e.g. oral, intravenous, subcutaneous)	
	**Dose frequency**	The number of times a treatment is administered within a specific time period (e.g. twice per week, once per year)	
	**Duration of administration**	The amount of time needed to complete one administration (e.g. 15 minutes, 1 hour)	
	**Dosage strength**	The strength of a treatment, which indicates the amount of active ingredient in each dosage (e.g. concentration of factor, concentration of vectors)	
	**Place of administration**	The geographical place where the treatment is administered (e.g. at home, hospital)	
	**Ease of administration**	The degree of ease to perform an administration	
	**Ease of product storage**	The degree of ease to store a treatment (e.g. the amount of storage space needed, temperature requirements)	
Follow-up	**Frequency of monitoring**	The number of times a patient has to visit a physician for follow-up on the effect of the treatment within a specific time period (e.g. once per month, once per year)	
Benefits	**Effect on factor level**	The effect on the amount of working clotting factor in the blood, delivered via factor replacement therapy or produced by the patient after gene therapy (often expressed in percentage, %, of normal levels)	
	**Effect on annual bleeding rate**	The effect of the treatment on the number of bleeding events per year	
	**Probability that prophylaxis can be stopped after treatment**	The chance that use of prophylactic factor replacement therapy can be stopped after treatment (expressed in percentage, %, of patients that can stop prophylaxis)	
	**Uncertainty regarding long-term benefits**	The degree of uncertainty that the effect of the treatment will be maintained after administration of the treatment (uncertainty may exist because of limited time that patients were followed-up after treatment administration, or because of limited numbers of patients treated with the treatment)	
Quality of Life	**Impact on daily life**	The impact of the treatment on daily activities	
	**Impact on participation in physical activity**	The impact of the treatment on the performance of physical activity (sports)	
	**Possibility to undergo major surgery**	The impact of the treatment on the possibility to undergo major surgery	
Risks	**Probability that liver inflammation will develop**	The chance that liver inflammation develops after treatment (expressed in percentage, %, of patients that develops liver inflammation)	
	**Uncertainty regarding long-term risks**	The degree of uncertainty regarding the side effects that can occur after administration of the treatment (uncertainty may exist because of limited time that patients were followed-up after treatment administration, or because of limited numbers of patients treated with the treatment)	
Spontaneously mentioned elements			

Supplementary material VI Script of the educational tool

**Screen 1: Introduction (number refers to timing of visual)**
**(1)** Welcome! My name is Anna, and I'm very happy to hear you want to participate in our study on patients' preferences regarding gene therapy for haemophilia. Before we start, I would like to make sure that you have all the information you need to form an opinion on this treatment, and to understand the questions in the survey. The information part will only take about 10 minutes!
**Screen 2: Introduction (continued)**
The information part is divided into 5 modules. First, we will discuss haemophilia and the current treatment options. Then you will receive more information about gene therapy, a treatment that is in clinical development and is not yet approved by the European Commission. Next, we will have a look at how gene therapy is administered, as well as the side effects and the therapeutic effects.<br>If you're ready to begin, please click start.
**Screen 3: Module 1 – information on haemophilia and the current treatment (1.1)**
**(1)** Haemophilia is a genetic disorder. This means that the disease is caused by an error located in the DNA that is present in genes. This error can arise spontaneously or can be passed along by one or both parents. **(2)** In haemophilia, the error is located in a gene for a clotting factor, or for short referred to as factor. Both Josh and Michael have haemophilia. In Josh the error is located in the gene for clotting factor IX which means he has haemophilia B. Michael has haemophilia A and the error is located in the gene for clotting factor VIII.
**Screen 4: Module 1 – information on haemophilia and the current treatment (1.2)**
**(1)** Because of this error, Josh and Michael are not able to produce the clotting factor or they do not produce enough of the correct clotting factor. This factor is crucial to stop bleeds, as it helps the clotting of blood. **(2)** Josh and Michael do not produce enough clotting factor which causes them to bleed for a longer time compared to people with the correct gene. In their case, bleeds can also happen spontaneously because they have a severe form of haemophilia and their body produces almost no functional clotting factor.
**Screen 5: Module 1 – information on haemophilia and the current treatment (1.3)**
**(1)** In haemophilia, the standard treatment supplements the patient's body with clotting factor in order to stop or prevent bleeds. Clotting factor cannot be taken via the mouth but has to be injected directly into a vein to reach the blood circulation. **(2)** Michael uses the treatment on-demand. This means he self-administers the clotting factor when a bleeding happens or when he knows that he will participate in an activity with a risk of bleeding. Josh treats himself in a 'prophylactic' manner. This means he self-administers the factor multiple times per week to keep the coagulation factor up to a level high enough to prevent bleeds. For haemophilia A patients this includes about two administrations per week and for haemophilia B patients one per week. However, the number of necessary injections per week can vary per individual. In addition to standard prophylactic therapy, it is now possible to receive long-acting clotting factors. These reduce the frequency of administration to once every 3 to 5 days for haemophilia A and once every 7 to 14 days for haemophilia B.
**Screen 6: Module 1 – information on haemophilia and the current treatment (1.4)**
**(1)** However, some patients may develop inhibitors that attack the administered factor. Inhibitors cause the clotting factor to not work so well anymore and can eventually lead to inefficacy of the treatment, meaning that bleeds can no longer be treated with the administration of factor.
**Screen 7: Overview**
We will now have a look at a new therapy for haemophilia that is in development, namely gene therapy.
**Screen 8: Module 2 – General information on gene therapy in haemophilia (2.1)**
**(1)** Both Josh and Michael will soon receive gene therapy. Let's have a look at how gene therapy works and the practical steps. **(2)** In general, gene therapy tries to correct the error in the genetic material so that the body contains the correct gene to produce the missing protein. In haemophilia, the goal of gene therapy is to deliver the correct gene of the clotting factor to the body. By doing this, the body will be able to produce the correct factor.
**Screen 9: Module 2 – General information on gene therapy in haemophilia (2.2)**
**(1)** How is this correct gene delivered to Josh and Michael? The correct gene is packaged in "a vector"; a modified, inactive virus. The virus itself is not infectious or functional. Only the casing of the virus remains, as a sort of taxi. The vector with the correct gene is administered once into a vein. The vector delivers the gene exclusively to the liver cells, since the liver is responsible for clotting factor production. **(2)** Once the vector has entered the cell, the new gene is added next to the genetic material that is already present. It will not alter patients' own genetic material which means that they can still pass on haemophilia to their children after receiving gene therapy.
**Screen 10: Overview**
Now that we know what gene therapy is, let's have a look at how it is used in patients.

Screen 11: Module 3 – Practical information on the treatment of haemophilia with gene therapy
So how is gene therapy administered to patients? **(1)** Josh and Michael receive gene therapy once via an infusion into a vein in the hospital. The administration takes about 30 minutes to 2 hours. When the vector delivers the correct gene to the liver cells, the liver cells themselves start to produce clotting factor. **(2)** After this one-time administration Josh and Michael need to visit the hospital for check-ups regularly during a period of about 1 year to monitor the factor level. After this year, long-term follow-up is necessary for which patients occasionally need to visit the hospital.
**Screen 12: Overview**
Now let's see what the results of gene therapy can be.
**Screen 13: Module 4 – Therapeutic effects of gene therapy in haemophilia (4.1)**
**(1)** These are Josh and Michael before they received gene therapy, and Harry who was also allowed to receive gene therapy. We are now going to fast-forward 2 months after treatment with gene therapy, to see what gene therapy could do for Josh, Michael and Harry. Just as in other patients treated with gene therapy, there is a large variability in results achieved by gene therapy between Josh, Michael and Harry. **(2)** Some patients no longer need factor injections after gene therapy and experience no or almost no bleeds anymore, like Josh. In contrast, other patients still occasionally need factor administration and still experience a few bleeds per year, like Michael. There are also patients that are in between these two outcomes and need a few factor infusions per year to prevent bleeds, like Harry.
**Screen 14: Module 4 – Therapeutic effects of gene therapy in haemophilia (4.2)**
Treatment with gene therapy is supposed to results in a factor concentration that is always on the same level. **(1)** This is in contrast to injections with clotting factor that result in a high factor concentration directly after injection, but a low concentration before the next injection. **(2)** This means that gene therapy may provide a stable factor level that is high enough to protect patients against bleeds, versus fluctuating factor levels with the factor injections. However, as we discussed before, there can be variability in results across patients.
**Screen 15: Module 4 – Therapeutic effects of gene therapy in haemophilia (4.3)**
**(1)** Currently we have clinical data from patients in clinical trials that cover 3 years on average for haemophilia A and 10 years for haemophilia B since gene therapy administration. **(2)** Due to the limited period of time that is covered by evidence from these clinical trials, it is uncertain for how long this therapy will results in enough production of clotting factor by the liver. In other words, it is unknown whether the therapy will provide a life-long effect. Nevertheless, patients can always start to use factor injections again when the effect of gene therapy is not sufficient to prevent and stop bleeds.
**Screen 16: Overview**
Now that we know more about the potential benefits of gene therapy, let's also have a look at the potential risks.
**Screen 17: Module 5 – Side effects of gene therapy in haemophilia (5.1)**
**(1)** After receiving gene therapy, some patients experience a light inflammation of the liver. Josh also experienced this. The inflammation can be caused by an immune response of the body towards the vector but cannot cause viral hepatitis like hepatitis C and does not cause noticeable symptoms. **(2)** When the light inflammation occurs, it needs to be treated with cortisone (corticosteroids) for multiple weeks to avoid a possible decrease of the achieved factor level.
**Screen 18: Module 5 – Side effects of gene therapy in haemophilia (5.2)**
**(1)** 30-50% of the population has had contact with the natural virus on which the vector was based and have become immune by developing antibodies against the used vectors. If these antibodies are already present, it could be possible the vectors may get destroyed before reaching the liver cells and the gene therapy will not be effective. Therefore, these patients currently do not qualify for treatment with gene therapy.
**Screen 19: Module 5 – Side effects of gene therapy in haemophilia (5.3)**
**(1)** Patients without pre-existing antibodies against the vector can be treated successfully with gene therapy. However, the treatment with that vector cannot be repeated because the patient will develop antibodies against the used vector. It is still unknown if patients can be treated with another type of vector if gene therapy would not work long enough. **(3)** In addition, there are still uncertainties or unknowns on the long-term safety of gene therapy. Gene therapy is a new treatment and there is limited experience with the treatment. Currently there are no signals that gene therapy may cause negative effects, but it is unknown what side effects may occur in the long term.
**Screen 22: End of educational tool**
You have completed the educational video. You are now ready to move on to the survey. Please go back to the survey screen and click on "Start choice questions".

Supplementary material VII Final survey (English)

PART 1 – CONSENT

Haemophilia patient survey

Thank you for wanting to participate in our survey on the use of gene therapy in haemophilia!

This survey is not compatible with mobile phones, please respond to this survey on your personal computer, laptop or tablet.

The survey consists of 5 parts: 1) Consent, 2) Information on you, 3) A video, 4) Choice questions, and 5) Survey evaluation questions.

Please read the study details below and confirm your participation by ticking the box at the bottom of the next page.

Study details

Researchers

Head of research:	Isabelle Huys<br>Clinical Pharmacology and Pharmacotherapy<br>University of Leuven<br>O&N II, Herestraat 49 - Box 521, 3000 Leuven, Belgium<br>Isabelle.huys@kuleuven.be<br>+3216330409
Contact person:	Eline van Overbeeke<br>Clinical Pharmacology and Pharmacotherapy<br>University of Leuven<br>O&N II, Herestraat 49 - Box 521, 3000 Leuven, Belgium<br>Eline.vanoverbeeke@kuleuven.be<br>+32 16 37 36 01

Funding acknowledgement: This study is part of the PREFER project. The Patient Preferences in Benefit-Risk Assessments during the Drug Life Cycle (PREFER) project has received funding from the Innovative Medicines Initiative 2 Joint Undertaking under grant agreement No 115966. This Joint Undertaking receives support from the European Union's Horizon 2020 research and innovation programme and EFPIA.

-NEXT-

Consent form – Version 2.0 – 05/03/20

Please read the terms below. If you agree to the terms, please tick the box at the bottom of this form to confirm your participation.

Terms:

- I have read the information sheet *[link to popup screen with information sheet]*, Version 2.0 – 05/03/20;
- I was given sufficient time to decide whether I am willing to participate in this study;
- I am aware that participating in this study is completely voluntary and deciding not taking part will not affect my current or future health care;
- I am aware that although I may be answering questions about medical products these responses will not lead to any change on my current health care nor to a drug prescription;
- I am aware that I can stop participating in this study at any time without affecting my current or future health care;
- I give permission for the researchers in the team to collect information about me in an anonymous way as described in the information sheet, including information on my personal treatment preferences, my health, age, gender, and living area, and enter this information into a secure electronic database for analysis;
- I give permission for my personal information to be stored for maximum 15 years and I am aware that I cannot request to delete this information once submitted;
- I give permission that the data of this study is shared among the PREFER partners (EU and non-EU countries) following the standards established by the European General Data Protection Regulation (GDPR), national and local laws;
- I give permission that my data can be used to address future research questions by the PREFER partners following the standards established by the European General Data Protection Regulation (GDPR), national and local laws;
- I give permission that the study results can later be used for publications as well as educational purposes.

If you agree to the terms, please tick the box below to confirm your participation:

☐ I agree with all terms listed above and hereby confirm my participation in this study [Participants can only continue with the survey if they accept the terms]

-NEXT-

POP-UP SCREEN X

Information sheet – Version 2.0 – 05/03/20

Haemophilia patient survey

Head of research: Isabelle Huys
Clinical Pharmacology and Pharmacotherapy
University of Leuven
O&N II, Herestraat 49 - Box 521, 3000 Leuven, Belgium
Isabelle.huys@kuleuven.be
+3216330409

Contact person: Eline van Overbeeke
Clinical Pharmacology and Pharmacotherapy
University of Leuven
O&N II, Herestraat 49 - Box 521, 3000 Leuven, Belgium
Eline.vanoverbeeke@kuleuven.be
+32 16 37 36 01

Dear Mr./Ms.,

You were invited by your physician or patient organisation to voluntarily participate in a study on opinions of patients on the use of gene therapy in haemophilia. Your opinion on current treatments and gene therapy will be asked. Before you confirm your participation in this study, we ask you to read this information sheet carefully. For any questions regarding this study, please contact the contact person mentioned at the top of this form.

What is the purpose of the study?
Using a survey, we want to investigate the opinion of haemophilia patients regarding the use of gene therapy and we want to understand what the main elements are that would influence their choice between a standard therapy and gene therapy.

Who is conducting the study?
The University of Leuven is the sponsor and controller of the study. The study is conducted by researchers at the University of Leuven in collaboration with the Université Libre de Bruxelles and RTI health solutions (a nonprofit organization with methodological expertise). Also physicians from the University Hospital of Leuven and Saint-Luc University Hospital are involved, as well as the haemophilia association, the AHVH. This study is part of the European 'The Patient Preferences in Benefit-Risk Assessments during the Drug Life Cycle' (PREFER) project, a collaboration between academic institutions and commercial companies from EU and non-EU countries. The project aims to assess when and how patient preferences on benefits and risks of treatments should be incorporated into decisions on medicinal products. The PREFER project has received funding from the Innovative Medicines Initiative 2 Joint Undertaking under grant agreement No 115966. This Joint Undertaking receives support from the European Union's Horizon 2020 research and innovation programme and the European Federation of Pharmaceutical Industries and Associations (EFPIA). For more information on the PREFER project and its partners, please visit www.imi-prefer.eu.

Is this study scientifically and ethically justified?
The Ethics Committee Research UZ / KU Leuven approved the study. Ethics committees verify if the rights of participants are respected by researchers during a study, if the balance between risks and benefits is beneficial for the participants and if the study is scientifically and ethically justified.

Do I have to participate?
Your participation is completely voluntary. You can refuse to participate. If you decide not to participate, this will not affect your current or future health care. Also participation in the study will not affect your current or future health care. There is no cost associated with participating in this survey and you will not receive any compensation to take part.

What is asked of me?
The survey consists of 5 parts: 1) Consent, 2) Information on you, 3) A video, 4) Choice questions, and 5) Survey evaluation questions. The survey will take about 20 minutes and can be filled in online. Information will be gathered on your personal treatment preferences, your health, age, gender, and living area. During the survey, information will be presented to you on the disease, current therapies and gene therapy.

How will my personal data be kept confidential?
The data collected during this preference study will be stored in the secured database Sharepoint at KU Leuven. The study will adhere to the national and local data protection laws. You can participate on an anonymous basis, meaning that researchers will not be able to discover your identity and that of other participants. Researchers from the University of Leuven will have direct access to the data and will share this data with other researchers. No reporting of your personal identifying information in reports or publications can happen. Your data will be processed in accordance with the European General Data Protection Regulation (GDPR) and the Belgian legislation (Belgian law 30 Jul 2018) on the protection of natural persons with regard to the processing of personal data.

How will my data be used?
Your data will be used to learn more about how patients perceive certain drugs and how they make choices between alternatives. In addition, your data may be used to answer future scientific research questions by PREFER partners (including commercial companies), support submission dossiers to regulators, and in the design of future studies. The results will be analyzed by researchers and health care professionals. The researchers intend to publish the results in scientific journals and disseminate them via presentations at congresses or meetings.

How will my data be shared and transferred?
The researchers may share the anonymous data with PREFER partners, regulatory authorities as well as with partners with whom it is working to jointly conduct scientific research in other countries. The data protection laws in these countries may be less protective than data protection laws in the European Economic Area (EEA). With regards to transfers from the EEA to other countries, including the U.S, standards will be followed that have been established by the European General Data Protection Regulation (GDPR), national and local laws.

How long will my personal data be stored?
Records containing your data will be retained at the study site (University of Leuven), as well as the central storage repository of the PREFER project (Uppsala University), for a period of maximum 15 years from the end of the study.

What rights do I have concerning my personal data?
As the data is gathered in an anonymous way, you will not be able to review, correct, update, restrict, object to processing, delete, or receive an electronic copy of the personal data you have provided. If you have questions about how we use your data, you can always contact the contact person for this study via: eline.vanoverbeeke@kuleuven.be. If you subsequently have any special concerns or wish to file a complaint, please contact the privacy team at KU Leuven via: privacy@kuleuven.be. You also have the right to lodge a complaint to the data protection authority in Belgium via e-mail: contact@apd-gba.be or phone: +32 (0)2 274 48 00.

Please contact the contact person mentioned at the top of this form for any questions regarding this study.

Thank you for your interest and participation!

PART 2 – INFORMATION ON YOU

Please fill out the information below. We would like to collect your answers on the questions below to learn more about you. The answers of all participants on these questions will be summarized as group characteristics in reports and publications.

D1. What is your age? _______________ [Drop down]

D2. What is your gender? [Single]

- ☐ Male
- ☐ Female
- ☐ Other
- ☐ I prefer not to answer

D3. Where do you live? [Single]

- ☐ Flanders
- ☐ Wallonia
- ☐ Brussels
- ☐ Other: _______________

D4. What type of haemophilia do you have? [Single]

- ☐ Haemophilia A
- ☐ Haemophilia B
- ☐ I don't have haemophilia A or B

[IF D1 <18 OR D3 "Other" OR D4 "I don't have haemophilia A or B" > EXIT survey] Thank you for your interest in this survey. Unfortunately, you are not the right fit for the survey. We appreciate your time and consideration. You have exited the survey.

D5. Do you have mild, moderate or severe haemophilia? [Single]

- ☐ Mild (Factor level without treatment is higher than 5 but lower than 40%)
- ☐ Moderate (Factor level without treatment is between 1-5%)
- ☐ Severe (Factor level without treatment is lower than 1%)

D6. What treatment are you on now? [Multiple]

- ☐ Prophylaxis (preventive treatment)
- ☐ Episodic (treatments when a bleeding occurs)
- ☐ Intensive treatment because of presence of inhibitors
- ☐ Hemlibra (Emicizumab)
- ☐ Gene therapy
- ☐ Other: ____

D7. [IF D6 "prophylaxis"] Number of prophylaxis injections per month: __________

D8. [IF D6 "prophylaxis" OR "episodic" OR "Intensive" OR "Hemlibra"] Are you currently having inhibitors against the factor replacement therapy? [Single]

- ☐ Yes
- ☐ No

D10. How satisfied are you with the treatment? [Single]

- ☐ Very satisfied
- ☐ Satisfied
- ☐ Neutral
- ☐ Unsatisfied
- ☐ Very unsatisfied

D11. How often do you have bleeds? [Single]

- ☐ More than 1 time per week
- ☐ 1-5 times per month
- ☐ Less than 12 times per year

D12. [IF D11 "More than 1 time per week"] How many bleedings do you have on average per week?

____

D13. [IF D11 "1-5 times per month"] How many bleedings do you have on average per month?

____

D14. [IF D11 "Less than 12 times per year"] How many bleedings do you have on average per year?

____

D15. How many of your joints are damaged? __________

D16. [IF D15 <0] How do you classify the overall severity of the damage? [Single]

- ☐ Mild
- ☐ Moderate
- ☐ Severe

D18. How would you estimate your knowledge on gene therapy to be? [Single]

- ☐ Very good
- ☐ Good
- ☐ Reasonable
- ☐ Bad
- ☐ Very bad

D19. Have you already discussed your treatment with gene therapy with a physician? [Single]

- ☐ Yes
- ☐ No

D20. [IF D19 "Yes"] What was the decision that you made with the physician? [Single]

- ☐ To receive gene therapy in a clinical trial
- ☐ To receive gene therapy outside of a clinical trial
- ☐ To not receive gene therapy
- ☐ No decision has been made so far

D21. What is your current employment status? [Single]

- ☐ Full-time employed
- ☐ Part-time employed
- ☐ Unemployed
- ☐ Retired
- ☐ Student

D23. How often do you have someone (like a family member, friend, hospital/clinic worker, or caregiver) help you read hospital materials? [Single]

- ☐ Always
- ☐ Often
- ☐ Sometimes
- ☐ Occasionally
- ☐ Never

D24. How often do you have problems learning about your medical condition because of difficulty understanding written information? [Single]

- ☐ Always
- ☐ Often
- ☐ Sometimes
- ☐ Occasionally
- ☐ Never

D25. How confident are you filling out medical forms by yourself? [Single]

- ☐ Extremely
- ☐ Quite a bit
- ☐ Somewhat
- ☐ A little bit
- ☐ Not at all

-NEXT-

We want to ask you a few questions about your quality of life.

Q1. Please click the ONE box that best describes your health TODAY.

MOBILITY

☐ I have no problems in walking about
☐ I have slight problems in walking about
☐ I have moderate problems in walking about
☐ I have severe problems in walking about
☐ I am unable to walk about

© *EuroQol Research Foundation. EQ-5D™ is a trade mark of the EuroQol Research Foundation*

-NEXT-

Q2. Please click the ONE box that best describes your health TODAY.

SELF-CARE

☐ I have no problems washing or dressing myself
☐ I have slight problems washing or dressing myself
☐ I have moderate problems washing or dressing myself
☐ I have severe problems washing or dressing myself
☐ I am unable to wash or dress myself

© *EuroQol Research Foundation. EQ-5D™ is a trade mark of the EuroQol Research Foundation*

-NEXT-

Q3. Please click the ONE box that best describes your health TODAY.

USUAL ACTIVITIES *(e.g. work, study, housework, family or leisure activities)*

☐ I have no problems doing my usual activities
☐ I have slight problems doing my usual activities
☐ I have moderate problems doing my usual activities
☐ I have severe problems doing my usual activities
☐ I am unable to do my usual activities

© EuroQol Research Foundation. EQ-5D™ is a trade mark of the EuroQol Research Foundation

-NEXT-

Q4. Please click the ONE box that best describes your health TODAY.

PAIN / DISCOMFORT

☐ I have no pain or discomfort
☐ I have slight pain or discomfort
☐ I have moderate pain or discomfort
☐ I have severe pain or discomfort
☐ I have extreme pain or discomfort

© EuroQol Research Foundation. EQ-5D™ is a trade mark of the EuroQol Research Foundation

-NEXT-

Q5. Please click the ONE box that best describes your health TODAY.

ANXIETY / DEPRESSION

☐ I am not anxious or depressed
☐ I am slightly anxious or depressed
☐ I am moderately anxious or depressed
☐ I am severely anxious or depressed
☐ I am extremely anxious or depressed

© EuroQol Research Foundation. EQ-5D™ is a trade mark of the EuroQol Research Foundation

-NEXT-

Q6.

- We would like to know how good or bad your health is TODAY.
- This scale is numbered from 0 to 100.
- 100 means the best health you can imagine.
  0 means the worst health you can imagine.
- Please click on the scale to indicate how your health is TODAY.

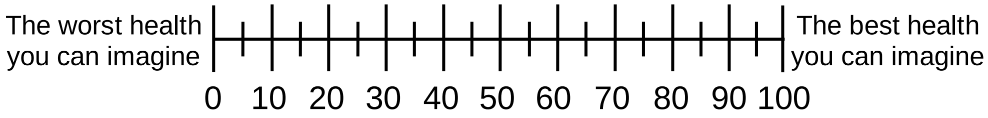

© EuroQol Research Foundation. EQ-5D™ is a trade mark of the EuroQol Research Foundation

-VOLGENDE-

PART 3 – VIDEO

Before we ask you questions about gene therapy, we would like to provide you with some information on haemophilia, current therapy and gene therapy through the educational video shown below. You may be familiar with some of the information presented, but we kindly ask you to complete all five modules before moving on to the next part of the survey.

To access the video, click on the "educational video" button below. The video will open on a separate screen.
When you have finished the five modules, you can return to this screen to continue Part 4, the choice questions.

Educational video

-START CHOICE QUESTIONS-

PART 4 – CHOICE QUESTIONS

Explanation on choice questions

In the next section we will ask you 7 “choice questions”. In these choice questions we will present you with two treatment options:

- Option A: this option reflects outcomes in patients that use factor replacement therapy in a prophylactic manner through regular intravenous administrations.
- Option B: this option reflects outcomes in patients treated with gene therapy through one intravenous administration.

The layout of the choice questions is given below. Four different features of the treatment options will be described. The outcomes of these features will be changed between the questions. For every choice question we ask you to indicate whether you would prefer to receive the factor replacement therapy or gene therapy based on the presented outcomes.

	**A.** Prophylactic factor replacement therapy	**B.** Gene therapy
Feature 1	Outcome 1 for option A	Outcome 1 for option B
Feature 2	Outcome 2 for option A	Outcome 2 for option B
Feature 3	Outcome 3 for option A	Outcome 3 for option B
Feature 4	Outcome 4 for option A	Outcome 4 for option B

-NEXT-

The four treatment features

The four treatment features that we will have a look at throughout the questions are: Annual bleeding rate, Chance to stop prophylaxis, Time that side effects have been studied, and Quality of life.

As it is crucial that you understand what is meant by these features we ask you to read the following descriptions very carefully before you go on to answer the choice questions.

-NEXT-

Treatment feature 1 - Annual bleeding rate:

With ‘Annual bleeding rate’ we mean the number of bleeding events per year while on prophylactic treatment or after receiving gene therapy. Annual bleeding rate is expressed in number of bleeds per year.

Annual bleeding rate is visually presented using blood droplets, where the number of blood droplets represents the number of bleeds per year. If the annual bleeding rate is 6 then this would be presented as:

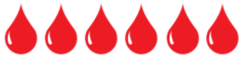

-NEXT-

Treatment feature 2 - Chance to stop prophylaxis:

With ‘Chance to stop prophylaxis’ we mean the chance that use of prophylactic factor replacement therapy can be stopped. Chance to stop prophylaxis is expressed in percentage (%) of patients that stop prophylaxis.

Patients that continue to use factor replacement therapy in a prophylactic manner will not be able to stop using this therapy; this is indicated as 0% chance to stop prophylaxis. Once patients are treated with gene therapy they have a chance to stop prophylaxis.

Chance to stop prophylaxis is visually presented using a figure that shows 10 figures of people. These 10 figures represent 100% of the patient population. Black figures show the part of the patient population that was able to stop using prophylaxis after treatment with gene therapy. Grey figures indicate the part of the patient population that was not able to stop prophylaxis. If the chance to stop prophylaxis is 90% (9 out of 10 people) then this would be presented as:

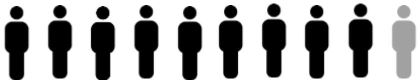

-NEXT-

Comprehension question

The picture on this page may help you think about how many people might be able to stop prophylaxis. Look at the picture of example 1 below.

- Each figure in the picture below represents 1 person
- There are 10 figures in the picture
- The figures shown in black indicate people who will be able to stop prophylaxis
- The figures shown in grey indicate people who will not be able to stop prophylaxis
- The more figures shown in black, the more likely it is that you would be able to stop prophylaxis

**Example 1**<br>4 of the figures are shown in black. That means that 4 out of 10 people (40%) will be able to stop prophylaxis.<br><br>6 of the figures are shown in grey. That means that 6 out of 10 people (60%) will not be able to stop prophylaxis.	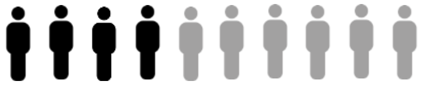

Please now look at example 2 and respond to the question below

**Example 2**	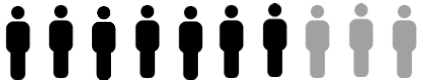

Question: If each figure in **example 2** is one person, how many people will be able to stop prophylaxis?

☐ 7 out of 10 (70%) [Correct answer]
☐ 3 out of 10 (30%)
☐ 5 out of 10 (50%)
☐ 2 out of 10 (20%)

[IF answer is not 70% or if question is skipped]
Remember, each figure in the picture represents one person. In the picture above, there are 10 figures, and 7 of them are black, while the rest are grey. This means that 7 people out of 10 people (or 70%) will be able to stop prophylaxis. The correct answer is 7 out of 10 (70%).

[IF answer is 70%]
You are correct. Each figure in the picture represents one person. In the picture above, there are 10 figures, and 7 of them are black, while the rest are grey. This means that 7 people out of 10 people (or 70%) will be able to stop prophylaxis. The correct answer is 7 out of 10 (70%).

-NEXT-

Treatment feature 3 - Time that side effects have been studied:

With ‘Time that side effects have been studied’ we mean the number of years that the side effects of prophylactic treatment or gene therapy have been studied in clinical trials and registries. If a medicine has been studied longer, more patients have used the medicine and patients have been followed-up for a longer time, so there is more information available about when and what type of side effects may occur.

The lower the number of years that a medicine has been studied, the more uncertainty there is regarding the long-term side effects (i.e. the side effects that will occur in the years after treatment). If a medicine for example has been studied for 3 years, we know what side effects may occur in those 3 years. The side effects may be the same for the following years, but they also might be somewhat different.

Time that side effects have been studied is visually presented using a bar of 50 years where the time that side effects have been studied is indicated in black. If the time that side effects have been studied would be 10 years then this would be presented as:

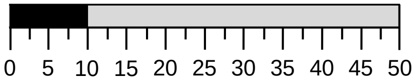

-NEXT-

Treatment feature 4 - Quality of life:

With “Quality of life” we mean the general health of a patient. Quality of life can be influenced by someone’s mobility, ability to care for themselves, ability to perform daily activities, pain or discomfort, and fear or depression. Quality of life is expressed using a scale that goes from 0 (worst possible quality of life) to 100 (best possible quality of life).

Quality of life is visually presented using a bar that goes from 0 to 100, with a black triangle indicating the achieved quality of life. If patients who take a therapy have a quality of life of 70, then this would be presented as:

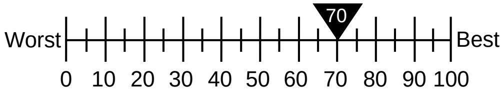

-NEXT-

[Choice questions - ANNUAL BLEEDING RATE SERIES]

[CA1 *<Annual bleeding rate level D>***] Choice question 1**

Suppose you are currently being treated with prophylactic factor replacement therapy and can switch to gene therapy. Results of gene therapy in other patients (that switched from prophylactic factor replacement therapy to gene therapy) showed that:

- It does **not reduce or increase the number of bleeds per year** and the number of bleeds remains at **6** bleeds per year on average.
- It allows **90% of patients** (9 out of 10 patients) **to stop prophylaxis** after getting the gene therapy
- **Side effects** of gene therapy have been **studied for 10 years**, while side effects of the prophylactic factor replacement therapy have been studied for 30 years
- It does **not reduce or increase quality of life** of patients and that the yearly average remains at **7**.

This situation is shown in the table below. In the table you can read the explanation of the different elements by hovering over the question mark button (wait 2 seconds - don't click!). Please tell us whether you would choose to remain on the prophylactic factor replacement therapy or get gene therapy by checking the box below your choice.

	**A.** Prophylactic factor replacement therapy	**B.** Gene therapy
Annual bleeding rate	6 bleeds per year 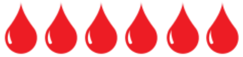	6 bleeds per year 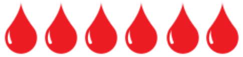
Chance to stop prophylaxis	0% (0 out of 10 patients stop prophylaxis) 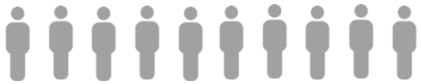	90% (9 out of 10 patients stop prophylaxis) 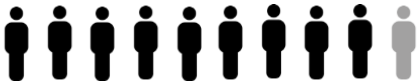
Time that side effects have been studied	30 years 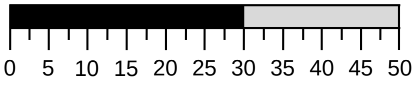	10 years 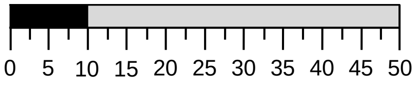
Quality of life	Quality of life of 70 out of 100 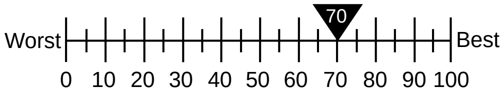	Quality of life of 70 out of 100 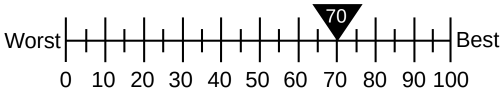

Please indicate your preferred choice: ☐ ☐

-NEXT-

[CA2 IF CA1 “prophylactic factor replacement therapy” <*Annual bleeding rate level B*>**] Choice question 2**

In the next couple of questions, the annual bleeding rate will be changed throughout the questions.
In the previous question, you said that you would prefer to remain on the prophylactic factor replacement therapy if with gene therapy the number of bleeds per year was 6, the chance to stop prophylaxis was 90%, side effects of gene therapy had been studied for 10 years, and quality of life was 70. What if the annual bleeding rate after gene therapy would be reduced to 3 bleeds per year, but all other features would remain the same? Please look at the table below and select the option you would prefer.

	**A.** Prophylactic factor replacement therapy	**B.** Gene therapy
Annual bleeding rate ?	6 bleeds per year	3 bleeds per year
Chance to stop prophylaxis ?	0% (0 out of 10 patients stop prophylaxis)	90% (9 out of 10 patients stop prophylaxis)
Time that side effects have been studied ?	30 years<br>0 5 10 15 20 25 30 35 40 45 50	10 years<br>0 5 10 15 20 25 30 35 40 45 50
Quality of life ?	Quality of life of 70 out of 100<br>70<br>Worst Best<br>0 10 20 30 40 50 60 70 80 90 100	Quality of life of 70 out of 100<br>70<br>Worst Best<br>0 10 20 30 40 50 60 70 80 90 100

Please indicate your preferred choice: ☐ ☐

-NEXT-

[CA3 IF CA2 “prophylactic factor replacement therapy” <*Annual bleeding rate level A*>**] Choice question 3**

In the last question, you said that you would prefer to remain on the prophylactic factor replacement therapy if the number of bleeds with gene therapy was 3 per year. What if the annual bleeding rate after gene therapy would be reduced to 1 bleed per year? Please look at the table below and select the option you would prefer.

	**A.** Prophylactic factor replacement therapy	**B.** Gene therapy
Annual bleeding rate	6 bleeds per year	1 bleed per year
Chance to stop prophylaxis	0% (0 out of 10 patients stop prophylaxis)	90% (9 out of 10 patients stop prophylaxis)
Time that side effects have been studied	30 years<br>0 5 10 15 20 25 30 35 40 45 50	10 years<br>0 5 10 15 20 25 30 35 40 45 50
Quality of life	Quality of life of 70 out of 100<br>Worst 70 Best<br>0 10 20 30 40 50 60 70 80 90 100	Quality of life of 70 out of 100<br>Worst 70 Best<br>0 10 20 30 40 50 60 70 80 90 100

Please indicate your preferred choice: ☐ ☐

-NEXT-

[CA4 IF CA2 “gene therapy” <*Annual bleeding rate level C*>**] Choice question 3**

In the last question, you said that you would prefer to receive gene therapy if the number of bleeds with gene therapy was 3 per year. What if the annual bleeding rate after gene therapy would be increased to 5 bleeds per year? Please look at the table below and select the option you would prefer.

	**A.** Prophylactic factor replacement therapy	**B.** Gene therapy
Annual bleeding rate ?	6 bleeds per year	5 bleeds per year
Chance to stop prophylaxis ?	0% (0 out of 10 patients stop prophylaxis)	90% (9 out of 10 patients stop prophylaxis)
Time that side effects have been studied ?	30 years<br>0 5 10 15 20 25 30 35 40 45 50	10 years<br>0 5 10 15 20 25 30 35 40 45 50
Quality of life ?	Quality of life of 70 out of 100<br>Worst 70 Best<br>0 10 20 30 40 50 60 70 80 90 100	Quality of life of 70 out of 100<br>Worst 70 Best<br>0 10 20 30 40 50 60 70 80 90 100

Please indicate your preferred choice: ☐ ☐

-NEXT-

[CA5 IF CA1 “gene therapy” <*Annual bleeding rate level F*>**] Choice question 2**

In the next couple of questions, the annual bleeding rate will be changed throughout the questions.
In the previous question, you said that you would prefer to receive gene therapy if with gene therapy the number of bleeds per year was 6, the chance to stop prophylaxis was 90%, side effects of gene therapy had been studied for 10 years, and quality of life was 70. What if the annual bleeding rate after gene therapy would be increased to 9 bleeds per year, but all other features would remain the same? Please look at the table below and select the option you would prefer.

	**A.** Prophylactic factor replacement therapy	**B.** Gene therapy
Annual bleeding rate	6 bleeds per year	9 bleeds per year
Chance to stop prophylaxis	0% (0 out of 10 patients stop prophylaxis)	90% (9 out of 10 patients stop prophylaxis)
Time that side effects have been studied	30 years<br>0 5 10 15 20 25 30 35 40 45 50	10 years<br>0 5 10 15 20 25 30 35 40 45 50
Quality of life	Quality of life of 70 out of 100<br>70<br>Worst Best<br>0 10 20 30 40 50 60 70 80 90 100	Quality of life of 70 out of 100<br>70<br>Worst Best<br>0 10 20 30 40 50 60 70 80 90 100

Please indicate your preferred choice: ☐ ☐

-NEXT-

[CA6 IF CA5 “gene therapy” *<Annual bleeding rate level G>*] **Choice question 3**

In the last question, you said that you would prefer to receive gene therapy if the number of bleeds with gene therapy was 9 per year. What if the annual bleeding rate after gene therapy would be increased to 11 bleeds per year? Please look at the table below and select the option you would prefer.

	**A.** Prophylactic factor replacement therapy	**B.** Gene therapy
Annual bleeding rate ?	6 bleeds per year	11 bleeds per year
Chance to stop prophylaxis ?	0% (0 out of 10 patients stop prophylaxis)	90% (9 out of 10 patients stop prophylaxis)
Time that side effects have been studied ?	30 years<br>0 5 10 15 20 25 30 35 40 45 50	10 years<br>0 5 10 15 20 25 30 35 40 45 50
Quality of life ?	Quality of life of 70 out of 100<br>Worst 70 Best<br>0 10 20 30 40 50 60 70 80 90 100	Quality of life of 70 out of 100<br>Worst 70 Best<br>0 10 20 30 40 50 60 70 80 90 100

Please indicate your preferred choice: ☐ ☐

-NEXT-

[CA7 IF CA5 “prophylactic factor replacement therapy” <*Annual bleeding rate level E*>**] Choice question 3**

In the last question, you said that you would prefer to remain on the prophylactic factor replacement therapy if the number of bleeds with gene therapy was 9 per year. What if the annual bleeding rate after gene therapy would be reduced to 7 bleed per year? Please look at the table below and select the option you would prefer.

	**A.** Prophylactic factor replacement therapy	**B.** Gene therapy
Annual bleeding rate ?	6 bleeds per year	7 bleeds per year
Chance to stop prophylaxis ?	0% (0 out of 10 patients stop prophylaxis)	90% (9 out of 10 patients stop prophylaxis)
Time that side effects have been studied ?	30 years<br>0 5 10 15 20 25 30 35 40 45 50	10 years<br>0 5 10 15 20 25 30 35 40 45 50
Quality of life ?	Quality of life of 70 out of 100<br>Worst 70 Best<br>0 10 20 30 40 50 60 70 80 90 100	Quality of life of 70 out of 100<br>Worst 70 Best<br>0 10 20 30 40 50 60 70 80 90 100

Please indicate your preferred choice: ☐ ☐

-NEXT-

[CA8 IF CA6 “gene therapy”**] Choice question 3b**

In the last question, you said that you would prefer to receive gene therapy if the number of bleeds with gene therapy was 11 per year. At what number of bleeds per year would you no longer have a preference between the two options? Please look at the table below and in the box below select the number of bleeds for gene therapy at which you would have no preference between the two options (at which they become equal to you).

	**A.** Prophylactic factor replacement therapy	**B.** Gene therapy
Annual bleeding rate ?	6 bleeds per year	? bleeds per year
Chance to stop prophylaxis ?	0% (0 out of 10 patients stop prophylaxis)	90% (9 out of 10 patients stop prophylaxis)
Time that side effects have been studied ?	30 years<br>0 5 10 15 20 25 30 35 40 45 50	10 years<br>0 5 10 15 20 25 30 35 40 45 50
Quality of life ?	Quality of life of 70 out of 100<br>Worst 70 Best<br>0 10 20 30 40 50 60 70 80 90 100	Quality of life of 70 out of 100<br>Worst 70 Best<br>0 10 20 30 40 50 60 70 80 90 100

Both options would become equal to me and I would have no preference if gene therapy would result in the following number of bleeds per year:
[Drop down between 11 and 100]

-NEXT-

[CA9 IF CA3 “prophylactic factor replacement therapy”**] Choice question 3b**

In the last question, you said that you would prefer to remain on the prophylactic factor replacement therapy if the number of bleeds with gene therapy was 1 per year. Please look at the table below. Would you accept gene therapy if gene therapy could result in 0 bleeds per year?

- ☐ Yes
- ☐ No, I would not accept gene therapy even if it could result in 0 bleeds per year

	**A.** Prophylactic factor replacement therapy	**B.** Gene therapy
Annual bleeding rate ?	6 bleeds per year	0 bleeds per year X
Chance to stop prophylaxis ?	0% (0 out of 10 patients stop prophylaxis)	90% (9 out of 10 patients stop prophylaxis)
Time that side effects have been studied ?	30 years<br>0 5 10 15 20 25 30 35 40 45 50	10 years<br>0 5 10 15 20 25 30 35 40 45 50
Quality of life ?	Quality of life of 70 out of 100<br>70<br>Worst Best<br>0 10 20 30 40 50 60 70 80 90 100	Quality of life of 70 out of 100<br>70<br>Worst Best<br>0 10 20 30 40 50 60 70 80 90 100

-NEXT-

[Choice questions - <u>CHANCE TO STOP PROPHYLAXIS SERIES</u>]

[Repeat 1 *<Chance to stop prophylaxis level D>***] Repetition of choice question 1**

In the first choice question of this survey you told us that you would prefer [INSERT “prophylactic factor replacement therapy” or “gene therapy” based on their answer on CA1]. This first question is presented again below.

First choice question

Suppose you are currently being treated with prophylactic factor replacement therapy and can switch to gene therapy. Results of gene therapy in other patients (that switched from prophylactic factor replacement therapy to gene therapy) showed that:

- It does **<u>not reduce or increase the number of bleeds per year</u>** and the number of bleeds remains at **6** bleeds per year on average.
- It allows **<u>90% of patients</u>** (9 out of 10 patients) **<u>to stop prophylaxis</u>** after getting the gene therapy
- **<u>Side effects</u>** of gene therapy have been **<u>studied for 10 years</u>**, while side effects of the prophylactic factor replacement therapy have been studied for 30 years
- It does **<u>not reduce or increase quality of life</u>** of patients and that the yearly average remains at **7.**

This situation is shown in the table below.

Please tell us whether you would choose to remain on the prophylactic factor replacement therapy or get gene therapy in this case.

	**A.** Prophylactic factor replacement therapy	**B.** Gene therapy
Annual bleeding rate ?	6 bleeds per year	6 bleeds per year
Chance to stop prophylaxis ?	0% (0 out of 10 patients stop prophylaxis)	90% (9 out of 10 patients stop prophylaxis)

Time that side effects have been studied	30 years<br>0 5 10 15 20 25 30 35 40 45 50	10 years<br>0 5 10 15 20 25 30 35 40 45 50
Quality of life	Score of 70 out of 100<br>70<br>Worst Best<br>0 10 20 30 40 50 60 70 80 90 100	Score of 70 out of 100<br>70<br>Worst Best<br>0 10 20 30 40 50 60 70 80 90 100

When you first answered this question, you chose [INSERT “prophylactic factor replacement therapy” or “gene therapy” based on their answer on CA1].
Do you still agree with that choice or would you like to change your choice?

☐ I still agree with my initial choice [Continue to question CP2 IF CA1 “prophylactic factor replacement therapy” or to question CP5 IF CA1 “gene therapy”]

☐ I would like to change my choice [REPEAT the ANNUAL BLEEDING RATE SERIES starting with question CA1, and after CA8 or CA9 continue to question CP2 IF CA1 “prophylactic factor replacement therapy” or to question CP5 IF CA1 “gene therapy” – save results from the first and second time that participants go through this series]

-NEXT-

[CP2 IF CA1 “prophylactic factor replacement therapy” <*Chance to stop prophylaxis level B*>**] Choice question 4**

In the next couple of questions, the chance to stop prophylaxis will be changed throughout the questions. In a previous question, you said that you would prefer to remain on the prophylactic factor replacement therapy if with gene therapy the number of bleeds per year was 6, the chance to stop prophylaxis was 90%, side effects of gene therapy had been studied for 10 years, and quality of life was 70. What if the chance to stop prophylaxis after gene therapy would increase to 100%, but all other features would remain the same? Please look at the table below and select the option you would prefer.

	**A.** Prophylactic factor replacement therapy	**B.** Gene therapy
Annual bleeding rate ?	6 bleeds per year	6 bleeds per year
Chance to stop prophylaxis ?	0% (0 out of 10 patients stop prophylaxis)	100% (10 out of 10 patients stop prophylaxis)
Time that side effects have been studied ?	30 years<br>0 5 10 15 20 25 30 35 40 45 50	10 years<br>0 5 10 15 20 25 30 35 40 45 50
Quality of life ?	Score of 70 out of 100<br>70<br>Worst Best<br>0 10 20 30 40 50 60 70 80 90 100	Score of 70 out of 100<br>70<br>Worst Best<br>0 10 20 30 40 50 60 70 80 90 100

Please indicate your preferred choice: ☐ ☐

-NEXT-

[CP4 [IF CP2 “gene therapy”] <*Chance to stop prophylaxis level C*>**] Choice question 5**

In the last question, you said that you would prefer to receive gene therapy if the chance to stop prophylaxis with gene therapy was 90%. What if the chance to stop prophylaxis after gene therapy would be 95%? Please look at the table below and select the option you would prefer.

	**A.** Prophylactic factor replacement therapy	**B.** Gene therapy
Annual bleeding rate	6 bleeds per year	6 bleeds per year
Chance to stop prophylaxis	0% (0 out of 10 patients stop prophylaxis)	95% (9.5 out of 10 patients stop prophylaxis)
Time that side effects have been studied	30 years	10 years
Quality of life	Quality of life of 70 out of 100	Quality of life of 70 out of 100

Please indicate your preferred choice: ☐ ☐

-NEXT-

[CP5 IF CA1 “gene therapy” <*Chance to stop prophylaxis level F*>**] Choice question 4**

In the next couple of questions, the chance to stop prophylaxis will be changed throughout the questions. In a previous question, you said that you would prefer to receive gene therapy if with gene therapy the number of bleeds per year was 6, the chance to stop prophylaxis was 90%, side effects of gene therapy had been studied for 10 years, and quality of life was 70. What if the chance to stop prophylaxis after gene therapy would be reduced to 80%, but all other features would remain the same? Please look at the table below and select the option you would prefer.

	**A.** Prophylactic factor replacement therapy	**B.** Gene therapy
Annual bleeding rate ?	6 bleeds per year	6 bleeds per year
Chance to stop prophylaxis ?	0% (0 out of 10 patients stop prophylaxis)	80% (8 out of 10 patients stop prophylaxis)
Time that side effects have been studied ?	30 years<br>0 5 10 15 20 25 30 35 40 45 50	10 years<br>0 5 10 15 20 25 30 35 40 45 50
Quality of life ?	Quality of life of 70 out of 100<br>Worst 70 Best<br>0 10 20 30 40 50 60 70 80 90 100	Quality of life of 70 out of 100<br>Worst 70 Best<br>0 10 20 30 40 50 60 70 80 90 100

Please indicate your preferred choice: ☐ ☐

-NEXT-

[CP6 IF CP5 “gene therapy” <*Chance to stop prophylaxis level G*>**] Choice question 5**

In the last question, you said that you would prefer to receive gene therapy if the chance to stop prophylaxis with gene therapy was 80%. What if the chance to stop prophylaxis after gene therapy would be 75%? Please look at the table below and select the option you would prefer.

	**A.** Prophylactic factor replacement therapy	**B.** Gene therapy
Annual bleeding rate ?	6 bleeds per year	6 bleeds per year
Chance to stop prophylaxis ?	0% (0 out of 10 patients stop prophylaxis)	75% (7.5 out of 10 patients stop prophylaxis)
Time that side effects have been studied ?	30 years<br>0 5 10 15 20 25 30 35 40 45 50	10 years<br>0 5 10 15 20 25 30 35 40 45 50
Quality of life ?	Quality of life of 70 out of 100<br>Worst 70 Best<br>0 10 20 30 40 50 60 70 80 90 100	Quality of life of 70 out of 100<br>Worst 70 Best<br>0 10 20 30 40 50 60 70 80 90 100

Please indicate your preferred choice: ☐ ☐

-NEXT-

[CP7 IF CP5 “prophylactic factor replacement therapy” *<Chance to stop prophylaxis level E>* **] Choice question 5**

In the last question, you said that you would prefer to remain on the prophylactic factor replacement therapy if the chance to stop prophylaxis with gene therapy was 80%. What if the chance to stop prophylaxis after gene therapy would be 85%? Please look at the table below and select the option you would prefer.

	**A.** Prophylactic factor replacement therapy	**B.** Gene therapy
Annual bleeding rate ?	6 bleeds per year 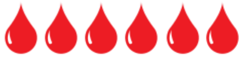	6 bleeds per year 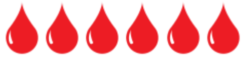
Chance to stop prophylaxis ?	0% (0 out of 10 patients stop prophylaxis) 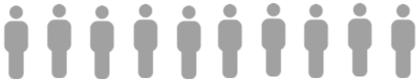	85% (8.5 out of 10 patients stop prophylaxis) 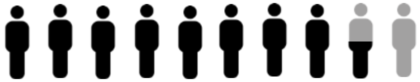
Time that side effects have been studied ?	30 years 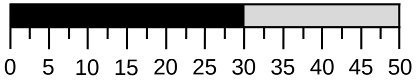	10 years 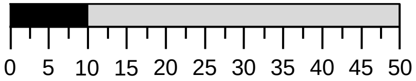
Quality of life ?	Quality of life of 70 out of 100 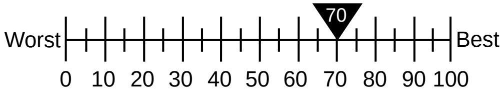	Quality of life of 70 out of 100 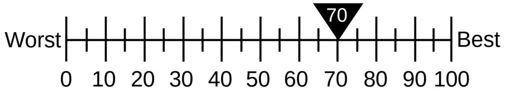

Please indicate your preferred choice: ☐ ☐

-NEXT-

[CP8a IF CP6 “gene therapy”] **Choice question 5b**

In the last question, you said that you would prefer to receive gene therapy if the chance to stop prophylaxis with gene therapy would be 75%. Please look at the table below. Would you accept gene therapy if the chance to stop prophylaxis with gene therapy would be 20%?

- ☐ Yes, I would still accept gene therapy if the chance to stop prophylaxis with gene therapy would be 20%
- ☐ No, I would not accept gene therapy

	**A.** Prophylactic factor replacement therapy	**B.** Gene therapy
Annual bleeding rate ?	6 bleeds per year 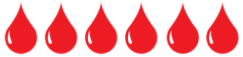	6 bleeds per year 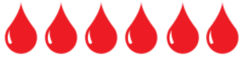
Chance to stop prophylaxis ?	0% (0 out of 10 patients stop prophylaxis) 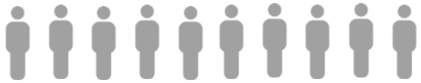	20% (2 out of 10 patients stop prophylaxis) 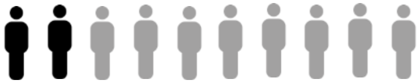
Time that side effects have been studied ?	30 years<br>0 5 10 15 20 25 30 35 40 45 50	10 years<br>0 5 10 15 20 25 30 35 40 45 50
Quality of life ?	Score of 70 out of 100<br>70<br>Worst Best<br>0 10 20 30 40 50 60 70 80 90 100	Score of 70 out of 100<br>70<br>Worst Best<br>0 10 20 30 40 50 60 70 80 90 100

-NEXT-

[CP8b IF CP8a “No”**] Choice question 5c**

At what chance to stop prophylaxis with gene therapy would you no longer have a preference between the two options? Please look at the table below and in the box below select the chance to stop prophylaxis with gene therapy (in percentages) at which you would have no preference between the two options (at which they become equal to you).

	**A.** Prophylactic factor replacement therapy	**B.** Gene therapy
Annual bleeding rate ?	6 bleeds per year	6 bleeds per year
Chance to stop prophylaxis ?	0% (0 out of 10 patients)	? %
Time that side effects have been studied ?	30 years (scale 0 5 10 15 20 25 30 35 40 45 50)	10 years (scale 0 5 10 15 20 25 30 35 40 45 50)
Quality of life ?	Score of 70 out of 100 (Worst 0 10 20 30 40 50 60 70 80 90 100 Best; marker 70)	Score of 70 out of 100 (Worst 0 10 20 30 40 50 60 70 80 90 100 Best; marker 70)

Both options would become equal to me and I would have no preference if gene therapy would result in the following chance to stop prophylaxis (%):
[Drop down between 20 and 75]

-NEXT-

[Choice questions - <u>QUALITY OF LIFE SERIES</u>]

[CQ2 IF CA1 “prophylactic factor replacement therapy” <*Quality of life level B*>**] Choice question 6**

In the next couple of questions, quality of life scores will be changed throughout the questions.
In a previous question, you said that you would prefer to remain on the prophylactic factor replacement therapy if with gene therapy the number of bleeds per year was 6, the chance to stop prophylaxis was 90%, side effects of gene therapy had been studied for 10 years, and quality of life was 70. What if the quality of life score with gene therapy would be increased to 80, but all other features would remain the same? Please look at the table below and select the option you would prefer.

	**A.** Prophylactic factor replacement therapy	**B.** Gene therapy
Annual bleeding rate ?	6 bleeds per year	6 bleeds per year
Chance to stop prophylaxis ?	0% (0 out of 10 patients stop prophylaxis)	90% (9 out of 10 patients stop prophylaxis)
Time that side effects have been studied ?	30 years<br>0 5 10 15 20 25 30 35 40 45 50	10 years<br>0 5 10 15 20 25 30 35 40 45 50
Quality of life ?	Quality of life of 70 out of 100<br>70<br>Worst Best<br>0 10 20 30 40 50 60 70 80 90 100	Quality of life of 80 out of 100<br>80<br>Worst Best<br>0 10 20 30 40 50 60 70 80 90 100

Please indicate your preferred choice: ☐ ☐

-NEXT-

[CQ3 IF CQ2 “prophylactic factor replacement therapy” <*Quality of life level A*>**] Choice question 7**

In the last question, you said that you would prefer to remain on the prophylactic factor replacement therapy if gene therapy could result in a quality of life score of 80. What if gene therapy could result in a quality of life score of 85? Please look at the table below and select the option you would prefer.

	**A.** Prophylactic factor replacement therapy	**B.** Gene therapy
Annual bleeding rate ?	6 bleeds per year 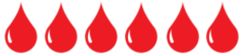	6 bleeds per year 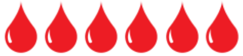
Chance to stop prophylaxis ?	0% (0 out of 10 patients stop prophylaxis) 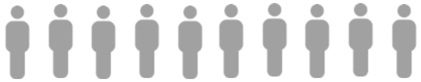	90% (9 out of 10 patients stop prophylaxis) 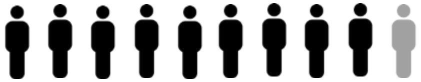
Time that side effects have been studied ?	30 years 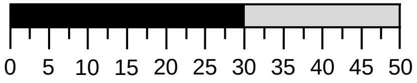	10 years 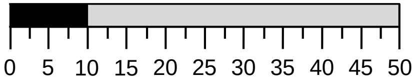
Quality of life ?	Quality of life of 70 out of 100 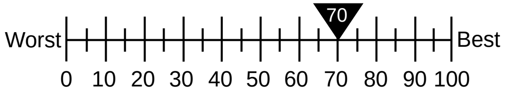	Quality of life of 85 out of 100 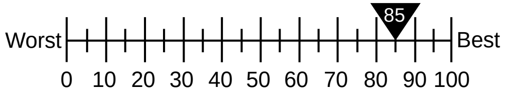

Please indicate your preferred choice: ☐ ☐

-NEXT-

[CQ4 IF CQ2 “gene therapy” <*Quality of life level C*>**] Choice question 7**

In the last question, you said that you would prefer to receive gene therapy if gene therapy could result in a quality of life score of 80. What if gene therapy could result in a quality of life score of 75? Please look at the table below and select the option you would prefer.

	**A.** Prophylactic factor replacement therapy	**B.** Gene therapy
Annual bleeding rate ?	6 bleeds per year	6 bleeds per year
Chance to stop prophylaxis ?	0% (0 out of 10 patients stop prophylaxis)	90% (9 out of 10 patients stop prophylaxis)
Time that side effects have been studied ?	30 years<br>0 5 10 15 20 25 30 35 40 45 50	10 years<br>0 5 10 15 20 25 30 35 40 45 50
Quality of life ?	Quality of life of 70 out of 100<br>70<br>Worst Best<br>0 10 20 30 40 50 60 70 80 90 100	Quality of life of 75 out of 100<br>75<br>Worst Best<br>0 10 20 30 40 50 60 70 80 90 100

Please indicate your preferred choice: ☐ ☐

-NEXT-

[CQ5 IF CA1 “gene therapy” <*Quality of life level F*>**] Choice question 6**

In the next couple of questions, quality of life scores will be changed throughout the questions.
In the last question, you said that you would prefer to receive gene therapy if with gene therapy the number of bleeds per year was 6, the chance to stop prophylaxis was 90%, side effects of gene therapy had been studied for 10 years, and quality of life was 70. What if the quality of life score with gene therapy would be reduced to 60, but all other features would remain the same? Please look at the table below and select the option you would prefer.

	**A.** Prophylactic factor replacement therapy	**B.** Gene therapy
Annual bleeding rate ?	6 bleeds per year	6 bleeds per year
Chance to stop prophylaxis ?	0% (0 out of 10 patients stop prophylaxis)	90% (9 out of 10 patients stop prophylaxis)
Time that side effects have been studied ?	30 years<br>0 5 10 15 20 25 30 35 40 45 50	10 years<br>0 5 10 15 20 25 30 35 40 45 50
Quality of life ?	Quality of life of 70 out of 100<br>Worst 70 Best<br>0 10 20 30 40 50 60 70 80 90 100	Quality of life of 60 out of 100<br>Worst 60 Best<br>0 10 20 30 40 50 60 70 80 90 100

Please indicate your preferred choice: ☐ ☐

-NEXT-

[CQ6 IF CQ5 “gene therapy” <*Quality of life level G*>**] Choice question 7**

In the last question, you said that you would prefer to receive gene therapy if gene therapy could result in a quality of life score of 60. What if gene therapy could result in a quality of life score of 55? Please look at the table below and select the option you would prefer.

	**A.** Prophylactic factor replacement therapy	**B.** Gene therapy
Annual bleeding rate	6 bleeds per year	6 bleeds per year
Chance to stop prophylaxis	0% (0 out of 10 patients stop prophylaxis)	90% (9 out of 10 patients stop prophylaxis)
Time that side effects have been studied	30 years	10 years
Quality of life	Quality of life of 70 out of 100	Quality of life of 55 out of 100

Please indicate your preferred choice: ☐ ☐

-NEXT-

[CQ7 IF CQ5 “prophylactic factor replacement therapy” <*Quality of life level E*>**] Choice question 7**

In the last question, you said that you would prefer to remain on the prophylactic factor replacement therapy if gene therapy could result in a quality of life score of 60. What if gene therapy could result in a quality of life score of 65? Please look at the table below and select the option you would prefer.

	**A.** Prophylactic factor replacement therapy	**B.** Gene therapy
Annual bleeding rate ?	6 bleeds per year	6 bleeds per year
Chance to stop prophylaxis ?	0% (0 out of 10 patients stop prophylaxis)	90% (9 out of 10 patients stop prophylaxis)
Time that side effects have been studied ?	30 years<br>0 5 10 15 20 25 30 35 40 45 50	10 years<br>0 5 10 15 20 25 30 35 40 45 50
Quality of life ?	Quality of life of 70 out of 100<br>Worst 70 Best<br>0 10 20 30 40 50 60 70 80 90 100	Quality of life of 65 out of 100<br>Worst 65 Best<br>0 10 20 30 40 50 60 70 80 90 100

Please indicate your preferred choice: ☐ ☐

-NEXT-

[CQ8a IF CQ6 "gene therapy"**] Choice question 7b**

In the last question, you said that you would prefer to receive gene therapy if gene therapy could result in a quality of life score of 55. Please look at the table below. Would you accept gene therapy if gene therapy could result in a quality of life score of 20?

- ☐ Yes, I would still accept gene therapy if it could result in a quality of life score of 20
- ☐ No, I would not accept gene therapy

	**A.** Prophylactic factor replacement therapy	**B.** Gene therapy
Annual bleeding rate ?	6 bleeds per year	6 bleeds per year
Chance to stop prophylaxis ?	0% (0 out of 10 patients stop prophylaxis)	90% (9 out of 10 patients stop prophylaxis)
Time that side effects have been studied ?	30 years<br>0 5 10 15 20 25 30 35 40 45 50	10 years<br>0 5 10 15 20 25 30 35 40 45 50
Quality of life ?	Score of 70 out of 100<br>70<br>Worst Best<br>0 10 20 30 40 50 60 70 80 90 100	Score of 20 out of 100<br>20<br>Worst Best<br>0 10 20 30 40 50 60 70 80 90 100

-NEXT-

[CQ8b IF CQ8a “No”**] Choice question 7c**

At what quality of life score would you no longer have a preference between the two options? Please look at the table below and in the box below select the quality of life score for gene therapy at which you would have no preference between the two options (at which they become equal to you).

	**A.** Prophylactic factor replacement therapy	**B.** Gene therapy
Annual bleeding rate ?	6 bleeds per year	6 bleeds per year
Chance to stop prophylaxis ?	0% (0 out of 10 patients stop prophylaxis)	90% (9 out of 10 patients stop prophylaxis)
Time that side effects have been studied ?	30 years<br>0 5 10 15 20 25 30 35 40 45 50	10 years<br>0 5 10 15 20 25 30 35 40 45 50
Quality of life ?	Score of 70 out of 100<br>70<br>Worst Best<br>0 10 20 30 40 50 60 70 80 90 100	Score of ?

Both options would become equal to me and I would have no preference if gene therapy would result in the following quality of life score :
[Drop down between 20 and 55]

-NEXT-

[CQ9a IF CQ3 “prophylactic factor replacement therapy”**] Choice question 7b**

In the last question, you said that you would prefer to remain on the prophylactic factor replacement therapy if gene therapy could result in a quality of life score of 85. Please look at the table below. Would you accept gene therapy if gene therapy could result in a quality of life score of 100 (best possible quality of life)?

☐ Yes

☐ No, I would not accept gene therapy even if it could result in a quality of life score of 100 (best possible quality of life)

	**A.** Prophylactic factor replacement therapy	**B.** Gene therapy
Annual bleeding rate ?	6 bleeds per year	6 bleeds per year
Chance to stop prophylaxis ?	0% (0 out of 10 patients stop prophylaxis)	90% (9 out of 10 patients stop prophylaxis)
Time that side effects have been studied ?	30 years	10 years
Quality of life ?	Score of 70 out of 100	Score of 100 out of 100

-NEXT-

[CQ9b IF CQ9a “yes”**] Choice question 7c**

At what quality of life score would you no longer have a preference between the two options? Please look at the table below and in the box below select the quality of life score for gene therapy at which you would have no preference between the two options (at which they become equal to you).

	**A.** Prophylactic factor replacement therapy	**B.** Gene therapy
Annual bleeding rate ?	6 bleeds per year	6 bleeds per year
Chance to stop prophylaxis ?	0% (0 out of 10 patients stop prophylaxis)	90% (9 out of 10 patients stop prophylaxis)
Time that side effects have been studied ?	30 years<br>0 5 10 15 20 25 30 35 40 45 50	10 years<br>0 5 10 15 20 25 30 35 40 45 50
Quality of life ?	Score of 70 out of 100<br>70<br>Worst Best<br>0 10 20 30 40 50 60 70 80 90 100	Score of ?

Both options would become equal to me and I would have no preference if gene therapy would result in the following quality of life score:
[Drop down between 85 and 100]

-NEXT-

PART 5 – SURVEY EVALUATION QUESTIONS

You completed all choice questions of our survey. We now would like to ask you six last questions on your opinion and understanding of our survey.

1. Throughout the choice questions, treatment options were described using four treatment features including quality of life. Quality of life was expressed using a scale that goes from 0 (worst possible quality of life) to 100 (best possible quality of life). What word would you use to describe a quality of life score of 70?
   - o Very Good
   - o Good
   - o Acceptable
   - o Poor
   - o Very Poor

2. How easy or difficult was it for you to understand the choice questions?

Very easy	Easy	Not easy nor difficult	Difficult	Very difficult
o	o	o	o	o

3. How easy or difficult was it for you to answer the choice questions?

Very easy	Easy	Not easy nor difficult	Difficult	Very difficult
o	o	o	o	o

4. Did the educational material (i.e. the video) help you understand the choice questions?

Very much	Moderately	Not sure	A little	Not at all
o	o	o	o	o

5. What did you think about the length of the questionnaire?

Just right	Manageable	Too long	Way too long
o	o	o	o

6. What could we have done to improve your understanding of the choice questions in this survey?

   ____

-NEXT-

Thank you for participating in this survey.
Your answers were well received.

References

1. Gene Therapy Clinical Trials Worldwide database [Internet]. Journal of Gene Medicine. 2018 [cited 2018 Dec 8]. Available from: http://www.abedia.com/wiley/search_results.php?TrialCountry=&CategoryMain=Monogenic+diseases&Vector=&GeneTypes=&Phase=Phase+III&Status=Open&FinalApprYear=&Submit=%A0%A0Search%A0%A0.
2. Batty P, Pasi KJ. Gene therapy trials for haemophilia: a step closer to a cure? Expert Review of Precision Medicine and Drug Development. 2019:1-4.
3. Nathwani AC, Tuddenham EGD, Rangarajan S, Rosales C, McIntosh J, Linch DC, et al. Adenovirus-Associated Virus Vector-Mediated Gene Transfer in Hemophilia B. The New England Journal of Medicine. 2011;365(25):2357-65.
4. Nathwani AC, Reiss UM, Tuddenham EGD, Rosales C, Chowdary P, McIntosh J, et al. Long-Term Safety and Efficacy of Factor IX Gene Therapy in Hemophilia B. New England Journal of Medicine. 2014;371(21):1994-2004.
5. George LA, Sullivan SK, Giermasz A, Rasko JEJ, Samelson-Jones BJ, Ducore J, et al. Hemophilia B Gene Therapy with a High-Specific-Activity Factor IX Variant. New England Journal of Medicine. 2017;377(23):2215-27.
6. Sullivan SK, Giermasz A, Samelson‐Jones BJ, Ducore JM, Teitel JM, Cuker A, et al. Investigational SPK‐9001: Adeno‐associated virus‐mediated gene transfer for hemophilia B ‐ persistent, stable factor IX activity at one year independent of downstream purification method. Haemophilia. 2018;24:209-18.
7. Miesbach W, Meijer K, Coppens M, Kampmann P, Klamroth R, Schutgens R, et al. Gene therapy with adeno-associated virus vector 5-human factor IX in adults with hemophilia B. Blood. 2018;131(9):1022-32.
8. Leebeek F, Meijer K, Coppens M, Kampmann P, Klamroth, Schutgens R, et al. Reduction in annualized bleeding and factor IX consumption up to 2.5 years in adults with severe or moderate-severe haemophilia B treated with AMT-060 (AAV5-hFIX) gene therapy. Blood. 2018;132:92–5856.
9. Chowdary P, Shapiro S, Davidoff AM, Reiss U, Alade R, Brooks G, et al. A Single Intravenous Infusion of FLT180a Results in Factor IX Activity Levels of More Than 40% and Has the Potential to Provide a Functional Cure for Patients with Haemophilia B. ASH Annual Meeting2018.
10. Fabb SA, Dickson JG. Technology evaluation: AAV factor IX gene therapy, Avigen Inc. Curr Opin Mol Ther. 2000;2(5):601-6.
11. Manno CS, Pierce GF, Arruda VR, Glader B, Ragni M, Rasko JJ, et al. Successful transduction of liver in hemophilia by AAV-Factor IX and limitations imposed by the host immune response. Nat Med. 2006;12(3):342-7.
12. Jiang H, Pierce GF, Ozelo MC, de Paula EV, Vargas JA, Smith P, et al. Evidence of multiyear factor IX expression by AAV-mediated gene transfer to skeletal muscle in an individual with severe hemophilia B. Mol Ther. 2006;14(3):452-5.
13. Lu DR, Zhou JM, Zheng B, Qiu XF, Xue JL, Wang JM, et al. Stage I clinical trial of gene therapy for hemophilia B. Sci China B. 1993;36(11):1342-51.
14. Rangarajan S, Walsh L, Lester W, Perry D, Madan B, Laffan M, et al. AAV5–Factor VIII Gene Transfer in Severe Hemophilia A. New England Journal of Medicine. 2017:NEJMoa1708483-NEJMoa.
15. Pasi KJ, Rangarajan S, Kim B, Lester W, Perry D, Madan B, et al. Achievement of normal circulating factor VIII activity following Bmn 270 AAV5-FVIII gene transfer: interim, long-term efficacy and safety results from a phase 1/2 study in patients with severe hemophilia A. Blood. 2017;130:603.
16. Rangarajan S, Kim B, Lester W, Symington E, Madan B, Laffan M, et al. Achievement of normal factor VIII activity following gene transfer with valoctocogene roxaparvovec (BMN 270): Long‐term efficacy and safety results in patients with severe hemophilia A. Haemophilia. 2018;24:65.
17. High KA, George LA, Eyster E, Sullivan SK, Ragni MV, Croteau SE, et al. A Phase 1/2 Trial of Investigational Spk-8011 in Hemophilia a Demonstrates Durable Expression and Prevention of Bleeds. ASH Annual Meeting2018.
18. Nathwani AC, Tuddenham EGD, Chowdary P, McIntosh J, Lee D, Rosales C, et al. GO-8: Preliminary Results of a Phase I/II Dose Escalation Trial of Gene Therapy for Haemophilia a Using a Novel Human Factor VIII Variant. ASH Annual Meeting2018.
19. Powell JS, Ragni MV, White GC, 2nd, Lusher JM, Hillman-Wiseman C, Moon TE, et al. Phase 1 trial of FVIII gene transfer for severe hemophilia A using a retroviral construct administered by peripheral intravenous infusion. Blood. 2003;102(6):2038-45.
20. Roth DA, Tawa NE, Jr., O'Brien JM, Treco DA, Selden RF. Nonviral transfer of the gene encoding coagulation factor VIII in patients with severe hemophilia A. N Engl J Med. 2001;344(23):1735-42.

21. U.S. Food and Drug Administration. Gene Therapy as a Treatment Modality for Hemophilia. [cited 2019 Feb 12]. Available from: https://www.fda.gov/downloads/ForPatients/PatientEngagement/UCM628022.pdf.
22. Teal S, Brohan E, Hettema Y, Humphrey L, Willgoss T, Hudgens S, et al. Development and psychometric evaluation of a novel tool for assessing patient perception and preference for haemophilia treatment (HaemoPREF). Haemophilia. 2014;20(5):666-73.
23. Bonanad S, Schulz M, Gordo A, Spurden D, Cicchetti M, Cappelleri JC, et al. HaemoPREF: Further evaluation of patient perception and preference for treatment in a real world setting. Haemophilia. 2017;23(6):884-93.
24. Chaugule SS, Hay JW, Young G. Understanding patient preferences and willingness to pay for hemophilia therapies. Patient Prefer Adherence. 2015;9:1623-30.
25. Michelsen S, van Overbeeke E, Huys I. Review on gene therapy in hemophilia. To be published. 2018.
26. Brown TM, Pashos CL, Joshi AV, Lee WC. The perspective of patients with haemophilia with inhibitors and their care givers: preferences for treatment characteristics. Haemophilia. 2011;17(3):476-82.
27. Steen Carlsson K, Andersson E, Berntorp E. Preference-based valuation of treatment attributes in haemophilia A using web survey. Haemophilia. 2017;23(6):894-903.
28. Furlan R, Krishnan S, Vietri J. Patient and parent preferences for characteristics of prophylactic treatment in hemophilia. Patient Prefer Adherence. 2015;9:1687-94.
29. Mantovani LG, Monzini MS, Mannucci PM, Scalone L, Villa M, Gringeri A, et al. Differences between patients', physicians' and pharmacists' preferences for treatment products in haemophilia: a discrete choice experiment. Haemophilia. 2005;11(6):589-97.
30. Mohamed AF, Epstein JD, Li-McLeod JM. Patient and parent preferences for haemophilia A treatments. Haemophilia. 2011;17(2):209-14.
31. Scalone L, Mantovani LG, Borghetti F, Von Mackensen S, Gringeri A. Patients’, physicians’, and pharmacists’ preferences towards coagulation factor concentrates to treat haemophilia with inhibitors: results from the COHIBA Study. Haemophilia. 2009;15(2):473-86.
32. DiBenedetti DB, Coles TM, Sharma T, Pericleous L, Kulkarni R. Assessing patients' and caregivers' perspectives on stability of factor VIII products for haemophilia A: a web-based study in the United States and Canada. Haemophilia. 2014;20(4):e296-303.
33. Costea I, Isasi R, Knoppers BM, Lillicrap D. Haemophilia gene therapy: the patients' perspective. Haemophilia. 2009;15(5):1159-61.
34. Lock J, de Bekker-Grob EW, Urhan G, Peters M, Meijer K, Brons P, et al. Facilitating the implementation of pharmacokinetic-guided dosing of prophylaxis in haemophilia care by discrete choice experiment. Haemophilia. 2016;22(1):e1-e10.
35. Cavazzana-Calvo M, Hacein-Bey S, de Saint Basile G, Gross F, Yvon E, Nusbaum P, et al. Gene therapy of human severe combined immunodeficiency (SCID)-X1 disease. Science. 2000;288(5466):669-72.
36. Musso R, Santoro R, Coppola A, Marcucci M, Sottilotta G, Targhetta R, et al. Patient preference for needleless factor VIII reconstitution device: the Italian experience. Int J Gen Med. 2010;3:203-8.
37. Moia M, Mantovani LG, Carpenedo M, Scalone L, Monzini MS, Cesana G, et al. Patient preferences and willingness to pay for different options of anticoagulant therapy. Intern Emerg Med. 2013;8(3):237-43.
38. Wasserman J, Aday LA, Begley CE, Ahn C, Lairson DR. Measuring health state preferences for hemophilia: development of a disease-specific utility instrument. Haemophilia. 2005;11(1):49-57.
39. Barlow JH, Stapley J, Ellard DR. Living with haemophilia and von Willebrand's: a descriptive qualitative study. Patient Educ Couns. 2007;68(3):235-42.
